# Developmental Functions of miR156-Regulated *SQUAMOSA PROMOTER BINDING PROTEIN-LIKE (SPL)* Genes in *Arabidopsis thaliana*

**DOI:** 10.1371/journal.pgen.1006263

**Published:** 2016-08-19

**Authors:** Mingli Xu, Tieqiang Hu, Jianfei Zhao, Mee-Yeon Park, Keith W. Earley, Gang Wu, Li Yang, R. Scott Poethig

**Affiliations:** Department of Biology, University of Pennsylvania, Philadelphia, Pennsylvania, United States of America; Max Planck, GERMANY

## Abstract

Correct developmental timing is essential for plant fitness and reproductive success. Two important transitions in shoot development—the juvenile-to-adult vegetative transition and the vegetative-to-reproductive transition—are mediated by a group of genes targeted by miR156, SQUAMOSA PROMOTER BINDING PROTEIN (SBP) genes. To determine the developmental functions of these genes in *Arabidopsis thaliana*, we characterized their expression patterns, and their gain-of-function and loss-of-function phenotypes. Our results reveal that *SBP-LIKE* (*SPL*) genes in *Arabidopsis* can be divided into three functionally distinct groups: 1) *SPL2*, *SPL9*, *SPL10*, *SPL11*, *SPL13* and *SPL15* contribute to both the juvenile-to-adult vegetative transition and the vegetative-to-reproductive transition, with *SPL9*, *SP13* and *SPL15* being more important for these processes than *SPL2*, *SPL10* and *SPL11*; 2) *SPL3*, *SPL4* and *SPL5* do not play a major role in vegetative phase change or floral induction, but promote the floral meristem identity transition; 3) *SPL6* does not have a major function in shoot morphogenesis, but may be important for certain physiological processes. We also found that miR156-regulated *SPL* genes repress adventitious root development, providing an explanation for the observation that the capacity for adventitious root production declines as the shoot ages. miR156 is expressed at very high levels in young seedlings, and declines in abundance as the shoot develops. It completely blocks the expression of its *SPL* targets in the first two leaves of the rosette, and represses these genes to different degrees at later stages of development, primarily by promoting their translational repression. These results provide a framework for future studies of this multifunctional family of transcription factors, and offer new insights into the role of miR156 in *Arabidopsis* development.

## Author Summary

In *Arabidopsis*, miR156 acts by repressing the expression of 10 *SQUAMOSA PROMOTOR BINDING PROTEIN-LIKE* (*SPL*) genes. The phenotype of plants over-expressing miR156 demonstrates that these genes control many aspects of plant development and physiology, but the functions of individual miR156-regulated *SPL* genes, and how their expression is regulated by miR156, are largely unknown. We addressed these questions by determining the phenotypes of loss-of-function mutations in these genes individually and in combination, and by comparing the expression patterns and the phenotypes of miR156-sensitive and miR156-resistant reporters for these genes. Our results reveal the unique and shared functions of the members of this gene family, and demonstrate that miR156 plays different roles in the regulation of SPL gene expression at different times in development.

## Introduction

Shoot development in plants can be divided into several more-or-less discrete phases based on the character of the lateral organs produced during each phase [1,2]. These phases consist of a juvenile vegetative, adult vegetative, and a reproductive phase, along with transition periods during which the shoot produces organs of intermediate identity. miR156, and the closely related miRNA, miR157, are the master regulators of the transition from the juvenile to the adult phase of vegetative development (vegetative phase change) [3,4]. miR156/miR157 are expressed at high levels in organs produced early in shoot development, where they repress the expression of their targets, *SQUAMOSA PROMOTER BINDING PROTEIN (SBP)* transcription factors [3,5–9]. Vegetative phase change is initiated by a decline in the expression of miR156/157 and the consequent increase in the expression of *SBP* genes in newly formed organs [7]. These *SBP* genes promote the development of adult vegetative traits and also promote floral induction in some species [10].

Although this model of shoot development is supported by studies in a number of herbaceous and woody plants, a detailed understanding of the function of individual *SBP* genes in vegetative and reproductive phase change is still lacking. *Arabidopsis* has 16 *SBP-LIKE* (*SPL*) genes, 10 of which are targeted by miR156 [3,5,11–14]. These genes can be grouped into 5 clades based on the amino acid sequence of their conserved DNA binding domain—*SPL3/SPL4/SPL5*, *SPL9/SPL15*, *SPL2/SPL10/SPL11*, *SPL6* and *SPL13A/B* [12,13,15]. The phenotype of transgenic plants constitutively over-expressing miR156 reveals that, as a group, these genes control many aspects of *Arabidopsis* development and physiology, including the timing of vegetative phase change and floral induction, the rate of leaf initiation, shoot branching, anthocyanin and trichome production on the inflorescence stem, stress responses, carotenoid biosynthesis, and shoot regeneration in tissue culture and lateral root development [3,6,7,16–28]. However, the role of individual *SPL* genes in these processes is still poorly understood.

The function of individual *SPL* genes has been investigated primarily by characterizing the phenotypes of plants expressing miR156-resistant versions of these genes under the regulation of their own promoter, or the constitutively expressed Cauliflower Mosaic Virus *35S* promoter. These over-expression phenotypes suggest that *SPL2*, *SPL9*, *SPL10*, *SPL11*, *SPL13*, and *SPL15* control a variety of processes in root and shoot development [7,17,19,21,23,24,29–31] whereas *SPL3*, *SPL4* and *SPL5* primarily promote floral induction and/or floral meristem identity [3,16,18,32]. However, in most cases, it is unknown if this over-expression phenotype reflects the normal function of these genes because their loss-of-function phenotypes have not been characterized, or are not readily apparent. The best-characterized members of this family are *SPL9* and *SPL15*: over-expression of these genes accelerates vegetative phase change and delays the rate of leaf initiation, whereas loss-of-function mutations have the opposite phenotype [7,17,30,33]. Consistent with their sequence similarity, the *spl9 spl15* double mutant has a stronger phenotype than either single mutant, although this phenotype is relatively mild compared to the phenotype of plants over-expressing miR156 [33]. This indicates that other targets of miR156 also have important functions in vegetative phase change. However, it is unknown of these functions are shared more-or-less equally by all members of this gene family or are the property of one or a few genes. Over-expression of *SPL3* accelerates abaxial trichome production and produces early flowering, but a loss-of-function mutation in this gene has no obvious phenotype [3,32]. Similarly, plants over-expressing *SPL10* have a reduced rate of leaf initiation and undergo vegetative phase change precociously, but an *spl10* mutation has no vegetative phenotype [7,17]. The loss-of-function phenotypes of other members of this gene family remain to be determined.

Here we describe the temporal and spatial expression patterns of the transcripts of miR156-regulated *SPL* genes, the expression patterns of miR156-sensitive and miR156-resistant translational reporters for these genes, and the phenotypes loss-of-function mutations in these genes, individually, and in combination. These results provide a detailed picture of the function of miR156-regulated *SPL* genes in *Arabidopsis* and the role of miR156 in their regulation. In addition to defining the developmental functions of miR156, we show that translational repression is more important for the function of miR156 than previously thought.

## Results

### *SPL* genes are translationally repressed during vegetative development by miR156

**Fig 1:**
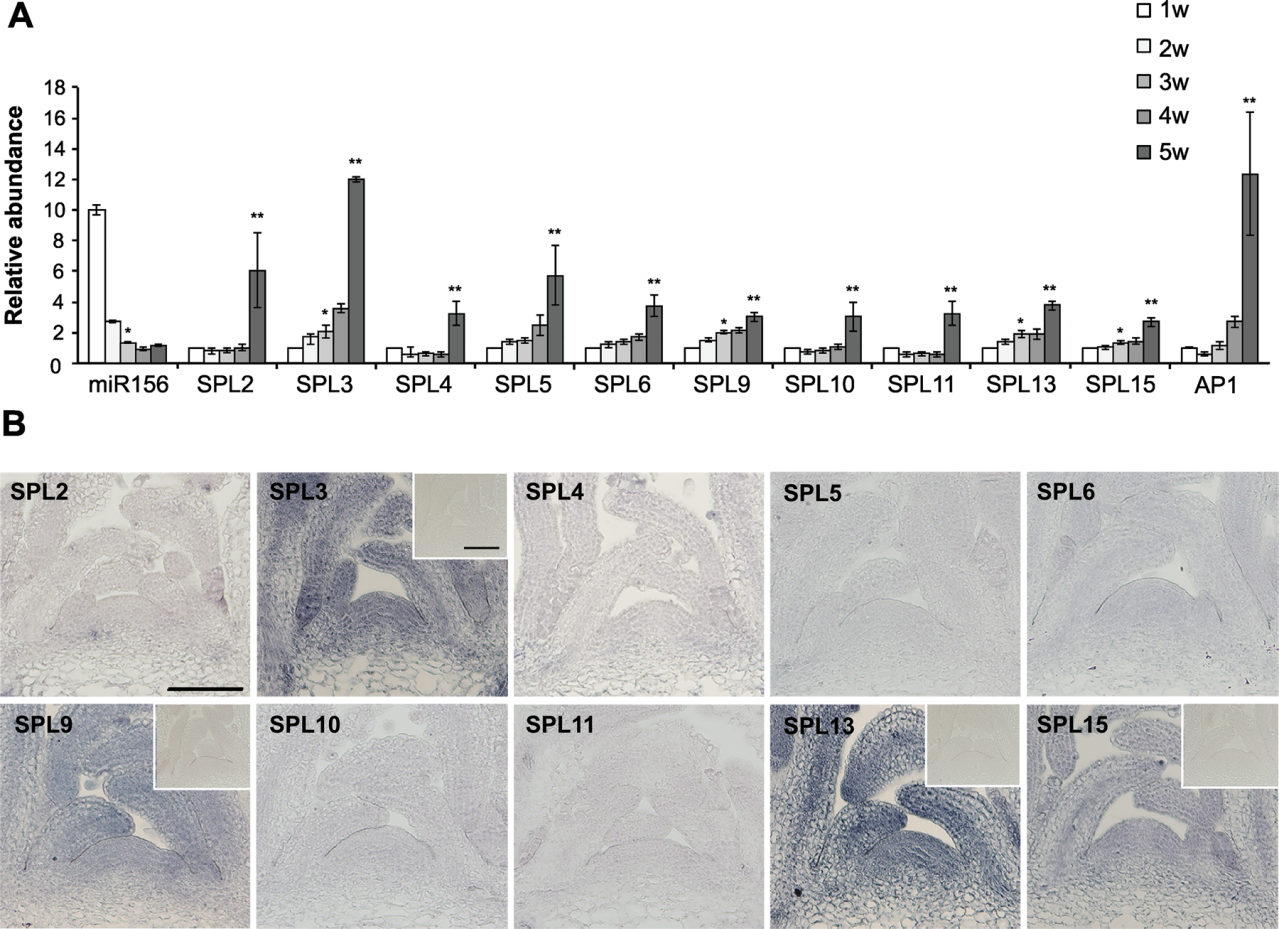
The abundance of miR156-regulated *SPL* transcripts in the shoot apices of wild-type Col grown in SD. (A) Relative abundance of miR156, miR156-regulated *SPL* and *AP1* transcripts in 1, 2, 3, 4 and 5 week-old shoot apices of wild-type Col. Values are normalized to the value for 1w and represent the mean ± SE from four biological replicates. The initial abundance of miR156 was arbitrarily set to 10. *Difference between 1w and 3w is significant, p<0.05. **Difference between 3w and 5w is significant, p<0.05. Student's T test. (B) *in situ* hybridization of miR156-regulated *SPL* transcripts in shoot apices of 3-week old wild-type Col. Samples were incubated for the same amount of time during the color reaction. *In situ* hybridization of the SPL3, SPL9, SPL13, and SPL15 probes to shoot apices of 3-week-old *sp3-1*, *spl9-4*, *spl13-1*, and *spl15-1* plants is shown in the small inserts, and demonstrates the lack of significant background hybridization. Scale bar = 50 μm.

The level of miR156 decreases dramatically in the shoot apex of *Arabidopsis* seedlings early in development [34]. To determine how this decrease affects the abundance of the transcripts of individual *SPL* genes, we used qRT-PCR to measure the level of miR156 and its direct targets in shoot apices over a 5-week period. Plants were grown in a 10hr light:14hr dark short day (SD) photoperiod to delay flowering, which occurred 5 weeks after planting based on the increase in the expression of the floral marker, *AP1* (Fig 1A). The level of miR156 decreased by about 90% from 1 week to 3 weeks, and declined very little after this time (Fig 1A). *SPL3*, *SPL9* and *SPL13* mRNA increased 2-fold from 1 to 3 weeks, whereas *SPL2*, *SPL4*, *SPL6*, *SPL10*, *SPL11* and *SPL15* transcripts increased less than 2-fold or remained constant during this period (Fig 1A). The transcripts of all of these genes increased significantly between 4 and 5 weeks, coincident with the increase in *AP1* expression (Fig 1A).

We then used *in situ* hybridization to examine the spatial distribution and the relative abundance of these *SPL* transcripts in the shoot apices of 3-week-old plants grown in SD, when the level of miR156 was near its minimum. *SPL3*, *SPL9*, *SPL13* and *SPL15* transcripts were uniformly expressed in the shoot apical meristem and in leaf primordia, but the transcripts of *SPL2*, *SPL4*, *SPL5*, *SPL6*, *SPL10* and *SPL11* could not be detected using the same approach (Fig 1B).

To obtain a comprehensive picture of the expression pattern of these *SPL* genes and the contribution of miR156 to this pattern, we produced transgenic plants expressing miR156-sensitive (sSPL) and miR156-resistant (rSPL) fusion proteins tagged with ß-glucoronidase (GUS). With the exception of *SPL9* and *SPL11*, GUS was inserted within a genomic fragment that extended from the gene upstream to the gene downstream of the *SPL* locus (S1 Fig). The *SPL10* and *SPL11* constructs do not include the 3'UTR and the sequence 3' of these genes. miR156-resistant constructs were generated by introducing mutations at the miR156 target site that did not alter the amino acid sequence (S1 Table). Only phenotypically wild-type lines were saved in the case of plants transformed with sSPL constructs, to ensure that the transgene was not over-expressed. For each construct, 6–12 lines homozygous for single insertion sites were identified, and these were then screened for GUS activity under LD conditions. Two lines that expressed GUS at an intermediate level relative to the range for each construct, and in the most frequent pattern, were saved for further analysis. Unless otherwise specified, sSPL and rSPL refer to these translational fusions.

**Fig 2:**
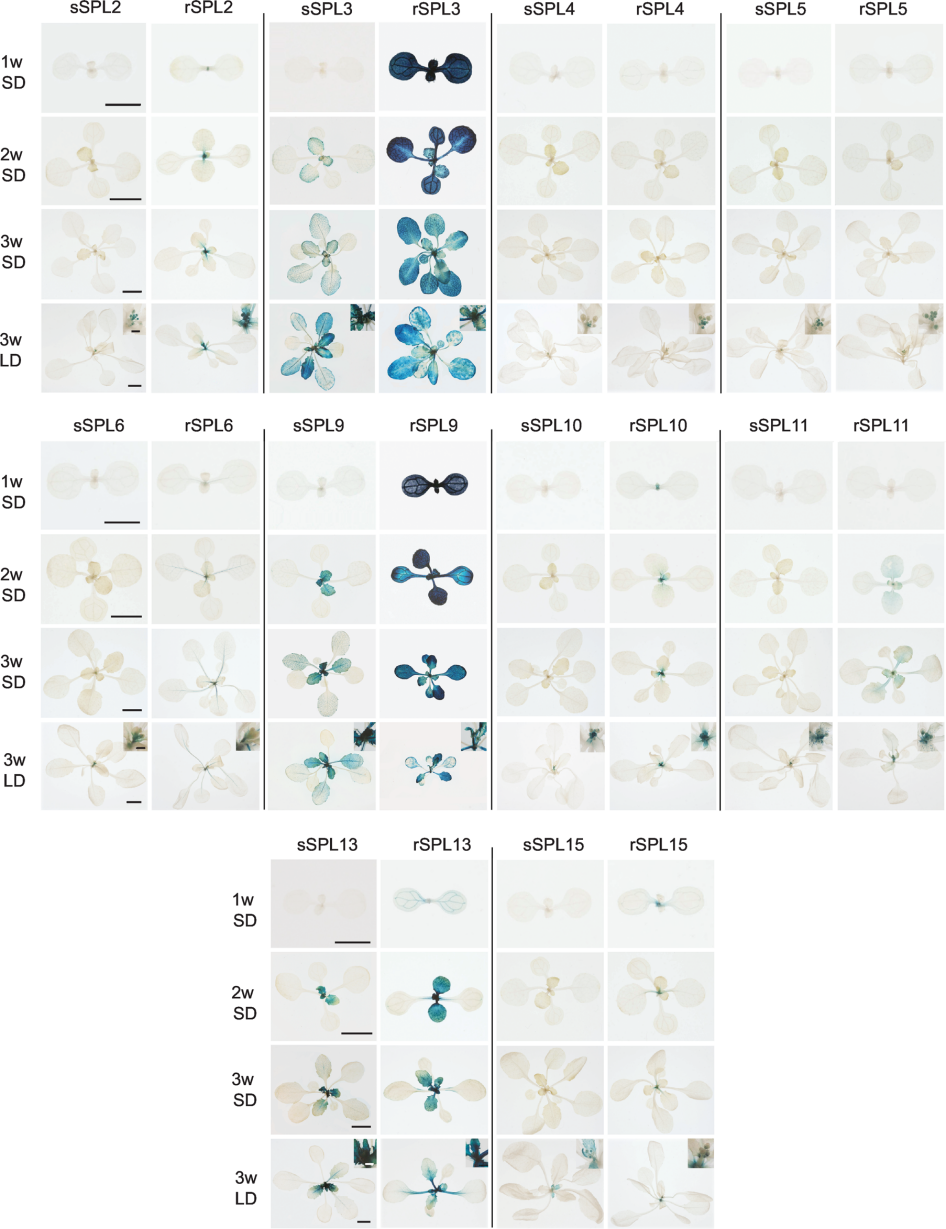
Expression of miR156-sensitive (sSPL) and miR156-resistant (rSPL) SPL-GUS fusion proteins in transgenic plants. Plants grown in SD were harvested 1, 2 and 3 weeks after planting; plants grown in LD were harvested at 3 weeks. The inserts in the 3w LD panels are a magnification of the inflorescence. Scale bars represent 2 mm for 1w and 2w, 5 mm for 3w, and 1 mm for the inserts.

The expression of sSPL and rSPL reporters was examined at 1, 2, and 3 weeks in plants growing in SD, and at 3 weeks in plants growing in LD (Fig 2). In SD, rSPL3 and rSPL9 were strongly expressed throughout leaf development in all rosette leaves, rSPL2, rSPL6, rSPL10, rSPL11, rSPL13 and rSPL15 were expressed in leaf primordia but not in fully expanded leaves, and rSPL4 and rSPL5 were undetectable. The miR156-sensitive versions of these constructs had a much more restricted expression pattern. With the exception of sSPL3, sSPL9, and sSPL13, all of these reporters were undetectable in rosette leaves (Fig 2). sSPL3, sSPL9, and sSPL13 were not expressed in leaves 1 and 2, but were expressed in all subsequent rosette leaves, although at a much lower level, and for a shorter time in leaf development than the rSPL reporters; this latter observation suggests that the abundance of miR156 increases as leaves expand, as has been reported in rice [35]. These results are consistent with the expression patterns of the transcripts these proteins (Fig 1), and indicate that most miR156-regulated *SPL* genes are transcribed throughout the vegetative phase of development in similar patterns, but are strongly and constitutively repressed during this phase by miR156. miR156 completely represses the expression of all of these genes in leaves 1 and 2, and represses their expression to varying degrees later in shoot development.

Three weeks after planting, the sSPL lines growing in SD were still vegetative, whereas the sSPL lines growing in LD were in the early stages of inflorescence development. At this stage, LD and SD plants had the same relatively low level of GUS expression in rosette leaves, but both the sSPL and the rSPL plants had GUS activity in the developing inflorescence (Fig 2). Some sSPL reporters were expressed at a lower level in than the corresponding rSPL reporter (e.g., sSPL2, sSPL10), but most sSPL and rSPL reporters were expressed at essentially the same level the inflorescence primordium. With the exception of sSPL15 and rSPL15, all reporters were expressed throughout inflorescence development and, in most cases, the expression patterns of the corresponding rSPL and sSPL reporters were nearly identical (S2 Fig). In contrast, sSPL15 and rSPL15 were only expressed during early stages of inflorescence development. These observations suggest that miR156 plays a minor role in the regulation of SPL activity during and after the floral transition, and that SPL15 is important for floral induction and/or the floral meristem identity transition, but not for later stages of inflorescence development.

### miR156-resistant *SPL*s accelerate vegetative phase change

**Fig 3:**
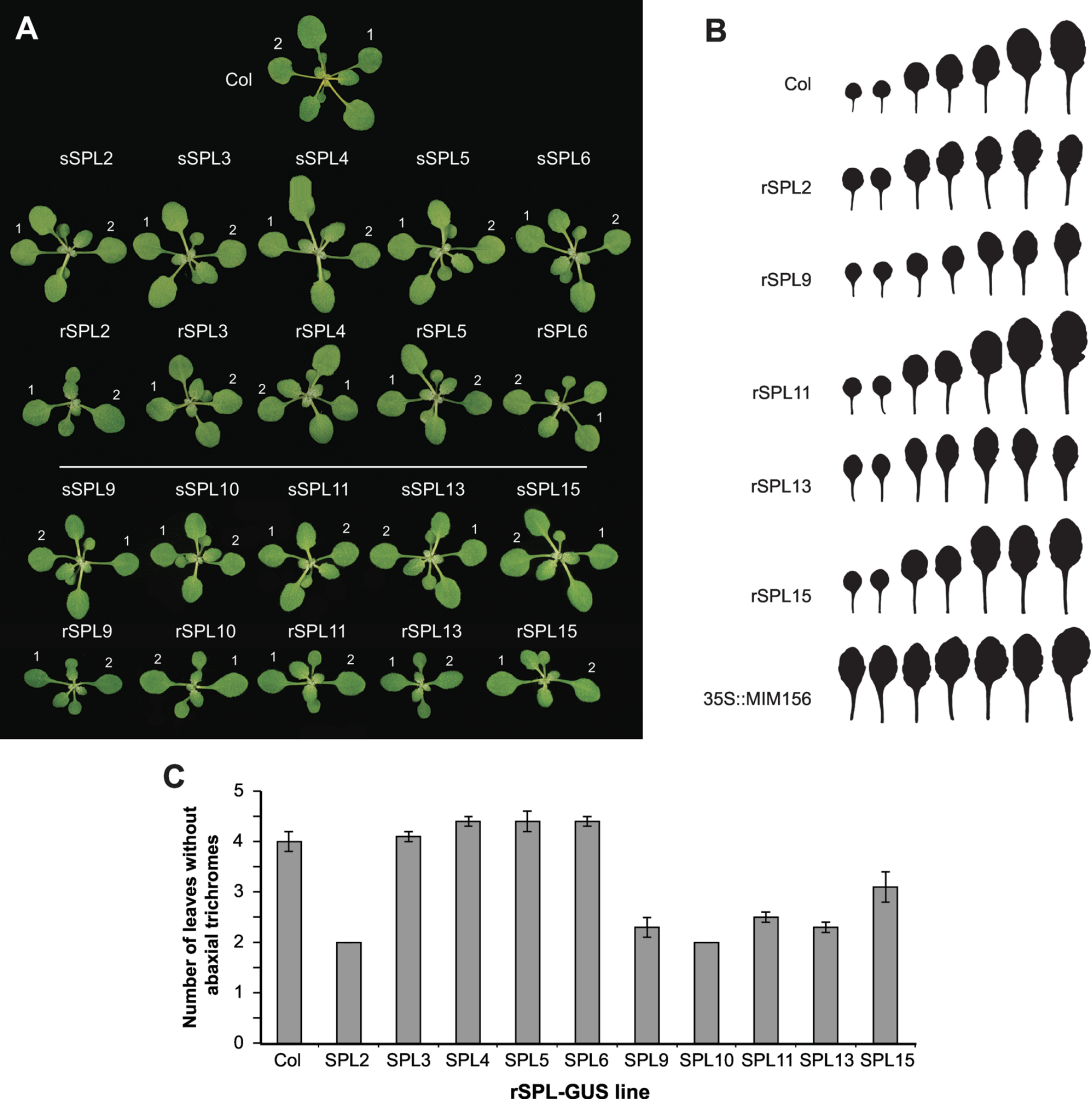
The vegetative phenotype of transgenic plants expressing sSPL-GUS and rSPL-GUS fusion proteins. (A) 17-day-old plants grown in SD. The first two rosette leaves are labelled. Scale bar = 10mm. (B) First 7 fully-expanded rosette leaves of Col, rSPL reporter lines, and a transgenic plant expressing a *35S*::*MIM156* target site mimic (SD). (C) The number of leaves without abaxial trichomes in Col and rSPL reporter lines.

Six of the rSPL lines (rSPL2, rSPL9, rSPL10, rSPL11, rSPL13 and rSPL15) had a phenotype that resembled the phenotype of plants with reduced levels of miR156, demonstrating that these GUS-fusion proteins are functional (Fig 3A and 3B). All of these lines had a reduced rate of leaf initiation, and the angle between the leaf blade and the petiole in leaves 1 and 2 was less acute than in the corresponding sSPL line (Fig 3A and 3B). In LD, these lines had fewer juvenile leaves (leaves without abaxial trichomes) than Col (Fig 3C). In contrast, rSPL3, rSPL4, rSPL5, and rSPL6 had little or no effect on the rate of leaf initiation and were not obviously different from Col (Fig 3A and 3C). SPL3, SPL4 and SPL5 are relatively small proteins, and we were concerned that the GUS fusion might disrupt their activity. To address this issue, we produced lines expressing rSPL3 without a GUS fusion. In comparison to a previously characterized *35S*::*rSPL3* line [3], which expresses SPL3 at approximately 1,700 times the normal level, these three lines had between 15–40 times the normal level of the *SPL3* transcript (S3 Fig). The *35S*::*rSPL3* line is early flowering and has slightly accelerated abaxial trichome production [3], but lines expressing *rSPL3* under the control of its endogenous regulatory sequences were not significantly different from Col with respect to abaxial trichome production, leaf number, or flowering time (Table 1, Experiment 1). These results suggest that *SPL2*, *SPL2*, *SPL9*, *SPL10*, *SPL11*, *SPL13* and *SPL15* promote vegetative phase change whereas *SPL3*, *SPL4*, *SPL5*, and *SPL6* do not contribute significantly to this process. These results also suggest that *SPL3* does not normally promote in floral induction in either LD or SD.

**Table 1:** The effect of *SPL* mutations on vegetative and reproductive development.

	Juvenile leaves	Rosette leaves	Cauline leaves	Days to flower	*n*
**Experiment 1**					
Long days					
Col	4.0 ± 0.1	11.9 ± 0.2	3.2 ± 0.1	27.5 ± 0.2	*23*
*r*SPL3 #2	4.4 ± 0.1	11.7 ± 0.2	3.3 ± 0.1	27.9 ± 0.3	*23*
*r*SPL3 #4	4.7 ± 0.1	12.3 ± 0.2	3.3 ± 0.1	27.9 ± 0.2	*24*
*r*SPL3 #10	4.8 ± 0.1	12.8 ± 0.2a	3.4 ± 0.1	28.6 ± 0.2^a^	*24*
Short days					
Col -0	8.5 ± 0.3	41.5 ± 0.9	N.D.	60.3 ± 0.5	*12*
*r*SPL3 #2	7.7 ± 0.2	42.5 ± 1.1	N.D.	59.9 ± 0.7	*12*
*r*SPL3 #4	6.8 ± 0.3	42.7 ± 0.7	N.D.	58.9 ± 0.7	*12*
*r*SPL3 #10	8.2 ± 0.3	39.2 ± 0.7	N.D.	60.2 ± 0.9	*12*
**Experiment 2**					
Long days					
Col	5.3 ± 0.1	12.2 ± 0.2	3.2 ± 0.1	30.5 ± 0.3	*43*
*spl3-1 spl4-1 spl5-1*	5.5 ± 0.1	12.0 ± 0.2	3.5 ± 0.1^a^	31.1 ± 0.2	*42*
Short days (10L:14D)					
Col	6.8 ± 0.1	42.0 ± 0.6	10.0 ± 0.2	65.3 ± 0.6	*24*
*spl3-1 spl4-1 spl5-1*	7.0 ± 0.2	45.9 ± 1.0^a^	9.0 ± 0.3	68.3 ± 1.4	*24*
Short days (8L:16D)					
Col	9.1 ± 0.1	47.7 ± 0.7	9.0 ± 0.3	75.0 ± 1.2	*36*
*spl3-1 spl4-1 spl5-1*	9.7 ± 0.8	49.2 ± 0.9	8.8 ± 0.8	76.9 ± 1.4	*41*
**Experiment 3**					
Long days					
Col	4.6 ± 0.1	11.0 ± 0.3	3.0 ± 0.1	28.0 ± 0.3	*23*
*spl2-1*	5.0 ± 0.1	11.2 ± 0.3	2.9 ± 0.1	27.7 ± 0.4	*21*
*spl9-4*	7.3 ± 0.1^a^	13.0 ± 0.2^a^	3.0 ± 0.1	28.0 ± 0.3	*23*
*spl10-2*	4.7 ± 0.1	10.8 ± 0.2	3.0 ± 0.1	26.7 ± 0.2	*23*
*spl11-1*	5.4 ± 0.2^a^	11.2 ± 0.3	3.5 ± 0.2^a^	26.8 ± 0.4	*21*
*spl13-1*	6.4 ± 0.2^a^	12.2 ± 0.3^a^	2.8 ± 0.1	28.0 ± 0.4	*22*
*spl15-1*	4.4 ± 0.1	11.1 ± 0.3	2.7 ± 0.1	26.3 ± 0.4	*21*
*spl13/15*	5.5 ± 0.1^a^	12.7 ± 0.4^a^	3.5 ± 0.2^a^^b^	28.5 ± 0.5	*24*
*spl9/15*	8.2 ± 0.2^a^^b^	16.5 ± 0.4^a^^b^	3.2 ± 0.1	30.2 ± 0.4^a^^b^	*21*
*spl2/9/11/15*	8.4 ± 0.2^b^	16.3 ± 0.4^b^	2.6 ± 0.1	28.5 ± 0.4	*24*
*spl9/13*	12.9 ± 0.3^d^	16.6 ± 0.4^b^	2.9 ± 0.1	29.5 ± 0.6^a^^b^	*21*
*spl9/13/15*	18.9 ± 0.3^e^	27.1 ± 0.6^e^	4.1 ± 0.1^a^^e^	33.0 ± 0.3^e^	*23*
*spl9/11/13/15*	18.9 ± 0.3^e^	28.1 ± 0.3^e^	4.7 ± 0.1^a^^e^	33.2 ± 0.3^e^	*24*
*spl2/9/13/15*	21.3 ± 0.4^f^	30.7 ± 0.4^f^	4.3 ± 0.1^a^^e^	33.6 ± 0.3^e^	*24*
*spl2/9/11/13/15*	24.0 ± 0.5^h^	36.3 ± 0.5^f^^h^	5.3 ± 0.2^h^	34.7 ± 0.4^f^	*23*
*35S*::*MIR156A*	28.0 ± 0.7^i^	39.7 ± 0.8^i^	5.3 ± 0.1^i^	35.0 ± 0.4^f^	*23*
Short days					
Col	7.0 ± 0.2	42.7 ± 0.7	N.D.	64.2 ± 0.6	*23*
*spl2-1*	7.3 ± 0.2	44.5 ± 0.3	N.D.	65.0 ± 1.0	*22*
*spl9-4*	11.3 ± 0.3^a^	46.0 ± 1.0	N.D.	60.8 ± 0.6	*18*
*spl10-2*	6.8 ± 0.2	39.5 ± 0.6	N.D.	60.0 ± 0.7	*23*
*spl11-1*	8.2 ± 0.3^a^	44.1 ± 1.1	N.D.	65.2 ± 0.8	*22*
*spl13-1*	9.2 ± 0.3^a^	45.0 ± 0.8^a^	N.D.	60.5 ± 0.9	*24*
*spl15-1*	7.7 ± 0.2	42.7 ± 1.2	N.D.	64.3 ± 0.5	*24*
*spl13/15*	9.4 ± 0.3^b^	44.8 ± 0.8	N.D.	59.6 ± 0.4	*24*
*spl9/15*	13.4 ± 0.4^a^^b^	53.0 ± 1.3^a^^b^	N.D.	62.7 ± 0.8	*24*
*spl2/9/11/15*	14.3 ± 0.4	56.7 ± 1.4	N.D.	69.1 ± 1.0^a^^c^	*24*
*spl9/13*	22.5 ± 0.4^d^	50.2 ± 1.0^a^^b^	N.D.	55.0 ± 0.8	*22*
*spl9/13/15*	31.5 ± 0.8^e^	59.8 ± 1.1^c^	N.D.	66.4 ± 0.5^a^^b^	*22*
*spl9/11/13/15*	35.5 ± 0.9^f^	64.9 ± 1.1^f^	N.D.	71.0 ± 1.4^f^	*24*
*spl2/9/13/15*	52.7 ± 1.5^g^	71.7 ± 1.3^g^	N.D.	70.6 ± 1.2^f^	*23*
*spl2/9/11/13/15*	56.9 ± 1.6^h^	76.7 ± 2.1^h^	N.D.	73.6 ± 1.4^f^	*22*
*35S*::*MIR156A*	74.1 ± 1.0^i^	86.5 ± 3.9^i^	N.D.	77.3 ± 1.1^i^	*23*
**Experiment 4**					
Long days					
Col	5.0 ± 0.2	10.7 ± 0.2	2.9 ± 0.1	27.8 ± 0.3	*21*
*spl9/13*	11.3 ± 0.2^a^	13.9 ± 0.3^a^	2.2 ± 0.2	28.2 ± 0.3	*19*
*spl9/13/15*	15.9 ± 0.3^e^	20.3 ± 0.4^e^	3.0 ± 0.1^e^	30.7 ± 0.3^a^	*21*
*spl2/9/13/15*	19.3 ± 0.5^f^	24.3 ± 0.6^f^	3.6 ± 0.1^f^	31.2 ± 0.4^a^	*23*
*spl2/9/11/13/15*	22.1 ± 0.5^h^	30.8 ± 0.6^h^	4.8 ± 0.1^h^	33.0 ± 0.3^f^	*24*
*spl2/3/5/9/11/13/15*	22.5 ± 0.4^h^	30.5 ± 0.5^h^	4.7 ± 0.2^h^	32.6 ± 0.3^f^	*21*
*spl2/9/10-3/11/13/15*	26.8 ± 0.8^i^	38.3 ± 0.6^i^^j^	4.9 ± 0.3^h^	36.3 ± 0.5^i^	*18*
*35S*::*MIR156A*	26.4 ± 0.5^i^	35.8 ± 0.8^i^	4.8 ± 0.1^h^	35.4 ± 0.3^i^	*22*
Short days					
Col	7.7 ± 0.2	39.6 ± 0.4	9.4 ± 0.3	58.6 ± 0.8	*18*
*spl9/13*	23.9 ± 1.7^a^	46.1 ± 0.6^a^	9.5 ± 0.2	53.6 ± 0.3	*22*
*spl9/13/15*	39.8 ± 0.6^e^	51.3 ± 0.8^e^	5.2 ± 0.2^k^	56.2 ± 0.7	*22*
*spl2/9/13/15*	46.9 ± 0.8^f^	57.8 ± 1.0^f^	5.0 ± 0.2^k^	55.4 ± 0.6	*23*
*spl2/9/11/13/15*	55.0 ± 0.5^h^	64.2 ± 0.7^h^	4.6 ± 0.1^k^	56.7 ± 0.2	*23*
*spl2/3/5/9/11/13/15*	53.6 ± 0.6^h^	64.5 ± 0.7^h^	4.7 ± 0.1^k^	58.3 ± 0.8	*23*
*spl2/9/10-3/11/13/15*	71.8 ± 0.8^i^^j^	83.0 ± 1.4^i^^j^	5.3 ± 0.2^k^	67.3 ± 1.3^a^^j^	*23*
*35S*::*MIR156A*	64.4 ± 0.7^i^	73.0 ± 1.2^i^	5.6 ± 0.1^k^	63.5 ± 0.8^a^	*24*

^a^Significantly greater than Col

^b^Significantly greater than parents

^c^Significantly greater than *spl9/15*

^d^Significantly greater than *spl2/9/11/15*

^e^Significantly greater than *spl9/13*

^f^Significantly greater than *spl9/13/15*

^g^Significantly greater that *spl9/11/13/15*

^h^Significantly greater than *spl2/9/13/15*

^i^Significantly greater than *spl2/9/11/13/15*

^j^Significantly greater than *35S*::*MIR156A*

^k^Significantly less than Col

N.D.: Not Determined

### Loss-of-function mutations in *SPL* genes

**Fig 4:**
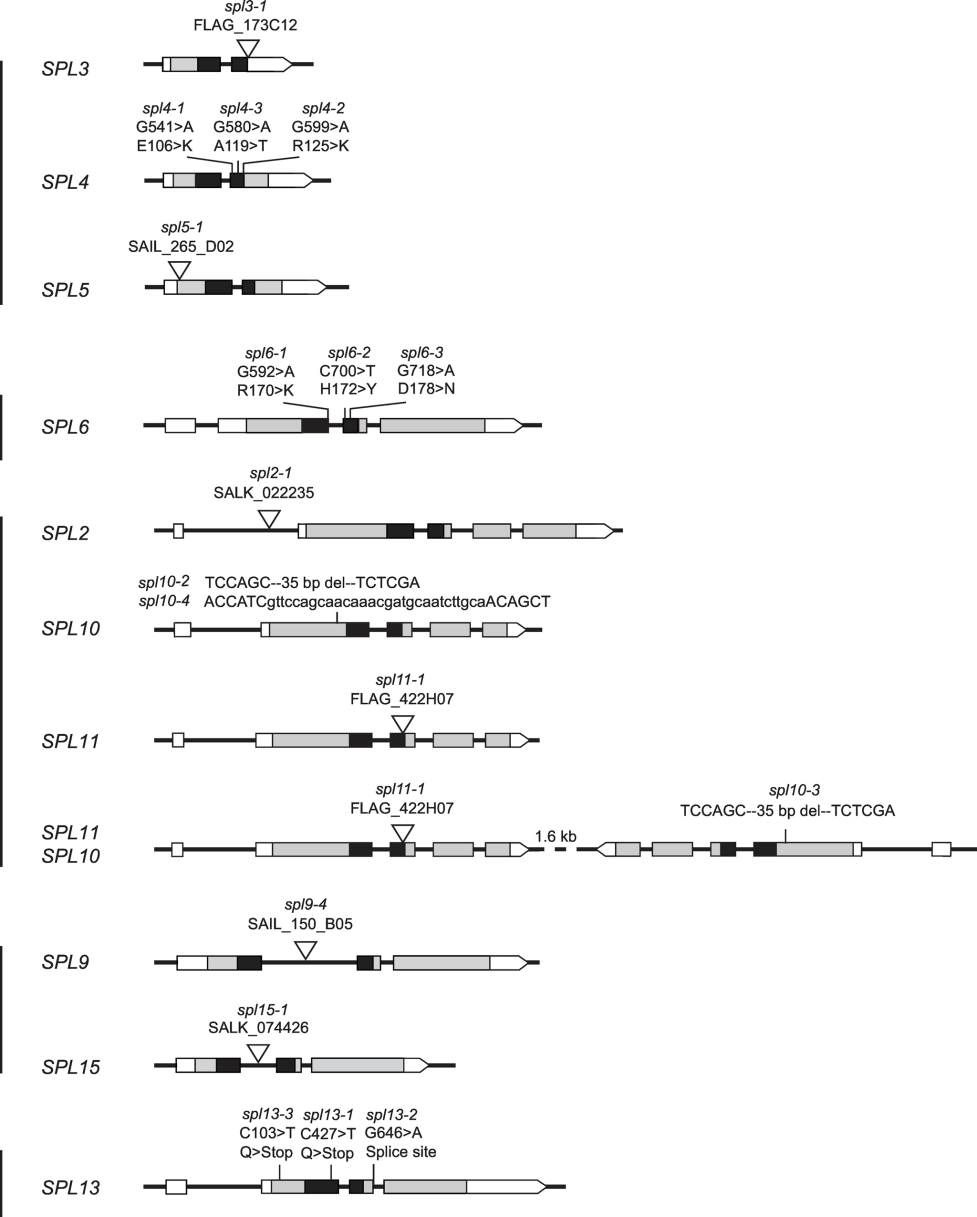
Loss-of-function alleles of *SPL* genes. White box = UTR, Grey box = transcribed region, Black box = SBP DNA binding domain. Genes in the same clade are indicated by vertical lines. The nucleotide and amino acid positions of point mutations are numbered from the translation start site.

The phenotype of plants expressing miR156-resistant SPL genes reveals the processes that these genes are capable of regulating, but does not necessarily reveal the processes in which they are actually involved because these transgenes may not be transcribed in a completely normal pattern or at a completely normal level due to their position in the genome or the presence of multiple T-DNAs at each insertion site [36,37]. To determine the normal functions of miR156-regulated *SPL* genes we therefore characterized the phenotypes of loss-of-function mutations in these genes (Fig 4). The mutations used for this analysis were generated by several different methods in several different ecotypes, and were introgressed into Col so that their phenotype could be compared. T-DNA (*spl2-1*, *spl5-1*, *spl9-2*, *spl9-4*, *spl15-1*, *spl15-2*) and CRISPR-Cas9 (*spl10-1*, *spl10-2*, *spl10-3*) alleles generated in Col were crossed to Col 3 times before use. T-DNA alleles generated in Ws (*spl3-1*, *spl11-1*), and EMS-induced alleles generated in Col (*spl4-1*, *spl4-2*) or Ler (*spl13-1*, *spl13-2*, *spl13-3*) were crossed to Col 6 to 8 times before use. All of the T-DNA mutations significantly reduced mRNA production (S4 Fig) and are likely to be null alleles. Different alleles of *spl9*, *spl13*, and *spl15* had similar effects on abaxial trichome production and produced similar phenotypes in combination with each other (S3 Table, Experiments 1, 2, and 3), so we only used one allele of these genes for the generation of multiple mutant lines. Although we only identified one allele of *SPL2*, *SPL3*, *SPL5* and *SPL11* that significantly affected the expression of these genes, all of these alleles were RNA null, had no obvious phenotype (*spl2*, *spl3*, *spl5*) or a very weak phase change phenotype (*spl11*) on their own, and had the expected phenotype in combination with other *spl* mutations. Consequently, we believe that this phenotype accurately reflects the function of these genes.

We were unable to identify T-DNA mutations that reduce the expression of *SPL4* so we used TILLing to identify mutations in this gene. Three missense mutations were selected for further study (Fig 4). All of these mutations affected highly conserved amino acids, but none had an obvious mutant phenotype. To determine if these mutations affect the function of *SPL4*, we took advantage of the observation that plants over-expressing an *SPL4* transcript without a miR156 target site (*35S*::*SPL4Δ*) are early flowering [3]. *35S*::*SPL4Δ* constructs containing each mutation (*35S*::*SPL4Δm*) were transformed into Col. A comparison of the flowering time of T1 plants transformed with *35S*::*SPL4Δ* and these *35S*::*SPL4Δm* constructs revealed that the G541-to-A mutation produced the largest reduction in SPL4 activity (S2 Table), so we used this allele (*spl4-1*) for all of the analyses described here.

Identifying mutations that block the activity of *SPL13* was problematic because there are two copies of this gene in Col, *SPL13A* (AT5G50570) and *SPL13B* (AT5G50670). These genes reside within a 33kb tandem duplication that is so recent that there are no polymorphisms between the duplicated segments. To determine if this duplication exists in other accessions of *Arabidopsis*, we used qPCR to compare the amounts of *SPL13* and *SPL9* DNA in Col and several other ecotypes. *SPL13* and *SPL9* were present in equal amounts in L*er*, Bak-2 and Voeran, but Col had twice as much *SPL13* as *SPL9* DNA (S5 Fig). This result suggests that the duplication in Columbia is relatively recent, and also suggests that the basal number of miR156-regulated *SPL* genes in *Arabidopsis* is 10, not 11, as is commonly reported. Importantly, the evidence that L*er* has a single *SPL13* gene meant that we could use mutations generated in this accession to obtain *spl13* loss-of-function alleles. 40 EMS-induced mutations of *SPL13* have been identified in L*er* (Martin et al, 2009). Two of these (*spl13-1*, *spl13-3*) introduce stop codons near the 5' end of the gene, and a second (*spl13-2*) is a mutation in the splice donor site in the third intron (Fig 4). We used *spl13-1* for all of our analyses because it was the most highly introgressed mutation at the start of these experiments.

A line containing T-DNA insertion in the first exon of *SPL10* has been identified [38], but this insertion does not reduce the *SPL10* transcript, and does not have an obvious phenotype, making it difficult to know if it has an effect on *SPL10* activity. Furthermore, it was impractical to recombine this mutation with mutations in its close paralog, *SPL11*, because *SPL10* and *SPL11* are only 1.6 kb apart. Consequently, mutations in *SPL10* were created using CRISPR-Cas9. *spl10-2* and *spl10-4* were generated in Col, and *spl10-3* was produced in a Col line containing *spl11-1* in order to generate an *spl10 spl11* double mutant. Sequencing of several potential off-target genes revealed no mutations in these genes. Fortuitously, *spl10-2* and *spl10-3* have an identical 35 bp deletion that produces a premature stop codon upstream of the SBP box. *spl10-4* has a 1 bp-deletion followed by 30 bp insertion, which also results in a premature stop codon upstream of the SBP-box domain (Fig 4).

We did not perform a detailed analysis of loss-of-function mutations of *SPL6* because we were unable to identify T-DNA insertions or EMS-induced mutations that unambiguously reduced the activity of this gene. TILLing produced 3 missense mutations in highly conserved amino acids that were predicted to have a damaging effect on protein function (Fig 4), but these mutations did not have an obvious phenotype. Over-expression of an *rSPL6* construct in transgenic plants under the regulation of the 35S promoter slightly accelerated vegetative phase change, but this effect was so weak that we did not believe that we would be able to assess the effect of these missense mutations on SPL function using this approach. The lack of a strong gain-of-function phenotype suggests that *SPL6* may not play a critical role in regulating vegetative morphogenesis.

Lines containing combinations of the mutations described above were generated by intercrossing mutant lines and identifying plants homozygous for the relevant alleles in F2 families, using allele-specific PCR (S4 Table). As we were generating these stocks we discovered that *spl2-1* is semi-sterile in heterozygous but not homozygous condition, and could not be recombined with *spl4-1*. This behaviour is characteristic of reciprocal translocations, and suggests that *spl2-1* contains a translocation with breakpoints near *SPL2* on chromosome 5 and *SPL4* on chromosome 1.

### miR156-regulated *SPLs* have overlapping as well as distinct roles in vegetative phase change

Phylogenetic analysis demonstrates that miR156-targetted *SPL* genes in *Arabidopsis* fall into 5 clades: *SPL3/SPL4/SPL5*, *SPL2/SPL10/SPL11*, *SPL9/SPL15*, *SPL6*, and *SPL13* [26,33,39] (Fig 4). We focused on *SPL2*, *SPL9*, *SPL10*, *SPL11*, *SPL13* and *SPL15* because the phenotype of plants expressing miR156-resistant versions of these genes indicated that they have a significant role in vegetative development. Because miR156-resistant transgenes and lack-of-function mutations in *SPL3*, *SPL4*, and *SPL5* did not have obvious phenotypes, we only conducted detailed analyses of the *spl3-1 spl4-1 spl5-1* (*spl3/4/5*) triple mutant. This triple mutant displayed no significant delay in vegetative phase change in LD and under two different SD conditions—10 hrs light:14 hrs dark and 8 hrs light:16 hrs dark (Table 1, Experiment 2). We performed all subsequent SD experiments using a 10 hrs light:14 hrs dark photoperiod because this photoperiod significantly delays flowering but does not produce a major delay in vegetative phase change.

Individually, *spl9-4*, *spl11-1* and *spl13-1* (hereafter, *spl9*, *spl11*, *spl13*) produced a small increase the number of juvenile leaves in both LD and SD, whereas *spl2-1*, *spl10-2*, and *spl15-1* (hereafter, *spl2*, *spl10*, *spl15*) had no obvious effect on juvenile leaf number (Table 1, Experiment 3). Plants mutant for more than one of these genes had much stronger phenotypes however. The strongest interaction we observed was between *spl9* and *spl13*. In LD, *spl9/13* had 6–8 more juvenile leaves and 3–5 more rosette leaves than Col, and in SD it had 15–16 more juvenile leaves and 6–7 more rosette leaves than Col (Table 1, Experiment 3, Experiment 4). The addition of *spl15* (*spl9/13/15*) produced a further delay in vegetative phase change and a larger increase in rosette leaf number (Table 1, Experiment 3, Experiment 4). In LD, *spl9/13/15* produced 11–14 more juvenile leaves and 9–16 more rosette leaves than Col, and in SD it produced 16–24 more juvenile leaves and 6–17 more rosette leaves than Col. These genotypes either had no effect on flowering time, or produced a very small delay in flowering, indicating that their effect on rosette leaf number is largely attributable to an increase in the rate of leaf initiation. These results suggest that *SPL9*, *SPL13* and *SPL15* strongly promote the juvenile phase and delay leaf initiation.

Mutations in *spl2*, *spl10* and *spl11* interacted weakly with each other and with *spl9*, *spl13* and *spl15* (S3 Table, Experiment 4). For example, the *spl2/9/11/15* quadruple mutant did not produce significantly more juvenile leaves or rosette leaves than *spl9/15* in either LD or SD (Table 1, Experiment 3). *spl9/11/13/15* was not significantly different from *spl9/13/15* in LD, and had only a slightly stronger phenotype than *spl9/13/15* in SD (Table 1, Experiment 3). *spl2* interacted more strongly with *spl9/13/15*: the *spl2/9/13/15* quadruple mutant produced significantly more juvenile leaves and rosette leaves than *spl9/13/15* in both LD and SD, although its phenotype was still much less severe than *35S*::*MIR156A* (Table 1, Experiments 3 and 4). Adding *spl11* to the *spl2/9/13/15* quadruple mutant (i.e. *spl2/9/11/13/15*) produced a further increase in juvenile leaf number and rosette leaf number, and adding both *spl10 and spl11* (*spl2/9/10/11/13/15*) produced a vegetative phenotype that was more severe than that of *35S*::*MIR156A* (Table 1, Experiment 4). The *spl2/3/5/9/11/13/15* sextuple mutant was not significantly different from *spl2/9/11/13/15* (Table 1, Experiment 4). These results provide additional evidence that *SPL9*, *SPL13* and *SPL15* play dominant roles in vegetative phase change, but reveal that *SPL2*, *SPL10* and *SPL11* also contribute to this developmental transition. Together, these 6 genes account for the effect of miR156 on vegetative phase change.

### miR156-regulated *SPLs* have distinct roles in flowering time and the specification of floral meristem identity

**Fig 5:**
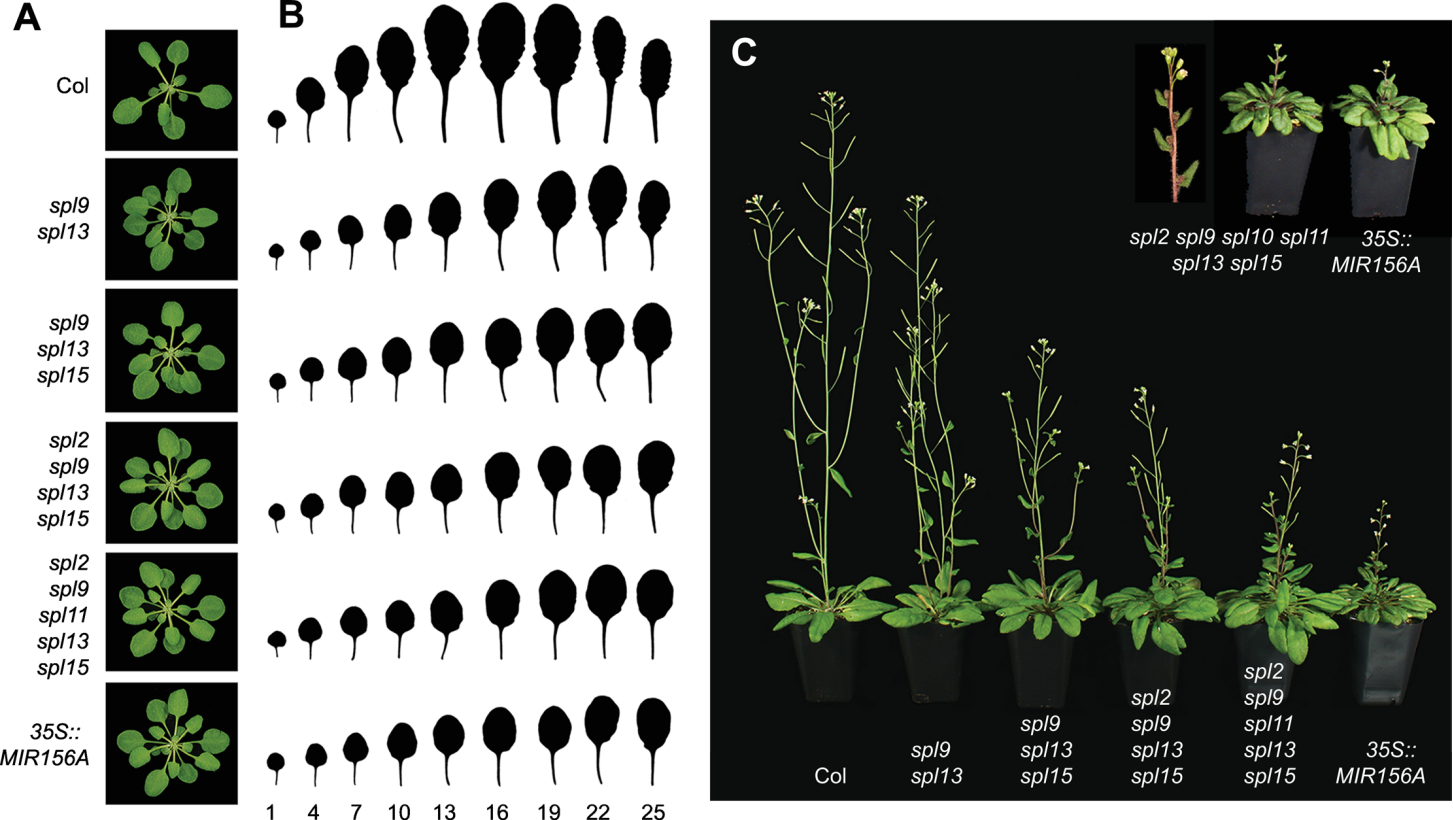
The phenotypes of *spl* mutant lines. (A) Rosettes of 21-day-old Col and *spl* mutants (SD). (B) The morphology of fully expanded rosette leaves of wild-type Col and *spl* mutants (SD). Leaves are numbered starting from the base of the rosette. (C) 5-week-old wild-type Col and *spl* mutants (LD). The inflorescence of an *spl2/9/10/11/13/15* plant is shown to demonstrate that the cauline leaves in this genotype subtend co-florescence buds, as is also the case for the *35S*::*MIR156A* line.

Leaf number cannot be used to measure flowering time in *spl* mutants because most of these mutations accelerate the rate of leaf initiation (Fig 5A) [17]. The major exception is *spl3/4/5*, which has no effect on the rate leaf initiation (S6 Fig). The effect of *spl* mutations on flowering time was therefore determined by recording the date of the first open flower. Many genotypes displayed small and sometimes statistically significant differences in flowering time relative to Col (Table 1, Experiments 2, 3 and 4), but is difficult to know if these differences are meaningful because we have observed similar variation between different stocks of Col; furthermore, some single and multiple mutant lines flowered earlier than Col, which is the opposite of the expected effect and is inconsistent with the phenotype of higher order mutant combinations and *35S*::*MIR156A*. However, certain combinations of mutations produced consistent effects on flowering time, which we believe accurately reflect the role of these genes in floral induction.

The phenotype of plants expressing *35S*::*MIR156A* reveals the overall contribution of miR156-regulated SPL genes to flowering time. This transgene consistently produced a 7–8 day delay in flowering in LD, but produced a more variable delay in flowering in SD, ranging from 5 to 12 days in different experiments (Table 1, Experiments 3 and 4). None of the single mutants flowered later than Col in either LD or SD. In LD, the most significant interactions occurred between *spl15* and other genotypes. Under these conditions, *spl9/15* flowered 2 days later than Col, and *spl9/13/15* flowered 4–5 days later than Col (Table 1, Experiments 3 and 4), In contrast, *spl9/13* only flowered 1 day later than Col, and *spl2/9/13* was not significantly different from *spl9/13 (*Table 1, Experiments 3 and 4; S3 Table, Experiment 4). The addition of *spl2*, *spl10* and *spl11* to *spl9/13/15* (*spl2/9/10/11/13/15*) produced a delay in flowering time equal to that of *35S*::*MIR156A* (Table 1, Experiment 4). We obtain different results in SD. In SD, most genotypes flowered earlier, or at approximately the same time as Col. *spl9/13/15* flowered 2 days later that Col in one experiment (Table 1, Experiment 3), but it did not flower significantly later than Col in a second experiment (Table 1, Experiment 4). Similarly, *spl2/9/11/13/15* flowered significantly later than Col and *spl9/13/15* in one experiment (Table 1, Experiment 3), but did not flower significantly later than these genotypes in a second experiment (Table 1, Experiment 4). Flowering was only consistently delayed in SD in the *spl2/9/10/11/13/15* hextuple mutant, which flowered later than *35S*::*MIR156A* in both LD and SD. Thus, *SPL2*, *SPL9*, *SPL10*, *SPL11*, *SPL13*, and *SPL15* all promote floral induction, and together explain the effect of *35S*::*MIR156A* on this process. These results also suggest that *SPL15* plays a more important role in floral induction than other *SPL* genes in LD.

*spl3/4/5* did not flower significantly later than Col in either LD or SD (Table 1, Experiment 2). Furthermore, adding *spl3* and *spl5* to *spl2/9/11/13/15* (*spl2/3/5/9/11/13/15)* did not produce an additional delay in flowering time beyond that observed for *spl2/9/11/13/15* (Table 1, Experiment 4). This result is consistent with the phenotype of the *rSPL3* transgenic lines (Table 1, Experiment 1), and demonstrates that these genes are not required for floral induction.

After floral induction, *Arabidopsis* makes several cauline leaves and co-florescence branches before transitioning to the floral phase of inflorescence development. This so-called "floral meristem identity" transition is promoted by *LFY* and *AP1* [40–46], which are bound by SPL3 and SPL9 *in vivo* and are up-regulated in plants over-expressing these genes [16,18]. The effect of *spl* mutations on the floral meristem identity transition was measured by counting the number of cauline leaves [45,47]. No single or double mutant had a significant effect on cauline leaf number in LD. However, *spl3/4/5* consistently produced approximately 1 extra cauline leaf (Table 1, Experiment 2) and *spl2/9/11/13/15* and other genotypes containing these mutations produced two extra cauline leaves in LD (Table 1, Experiment 4; Fig 5C), which was identical to the effect of *35S*::*MIR156A*. In SD, *spl9/13* produced the same number of cauline leaves as Col, but *35S*::*MIR156A* and other multiple mutants only produced 5 cauline leaves—4 less than the number of cauline leaves in Col (Table 1, Experiment 4). These results indicate that *SPL2*, *SPL3*, *SPL4*, *SPL5 SPL9*, *SPL10*, *SPL11*, *SPL13*, and *SPL15* promote the floral meristem identity transition in LD, and that *SPL2*, *SPL10*, *SPL11*, and/or *SPL15* inhibit this transition in SD.

### The effect of *spl* mutations on the expression of flowering genes

**Fig 6:**
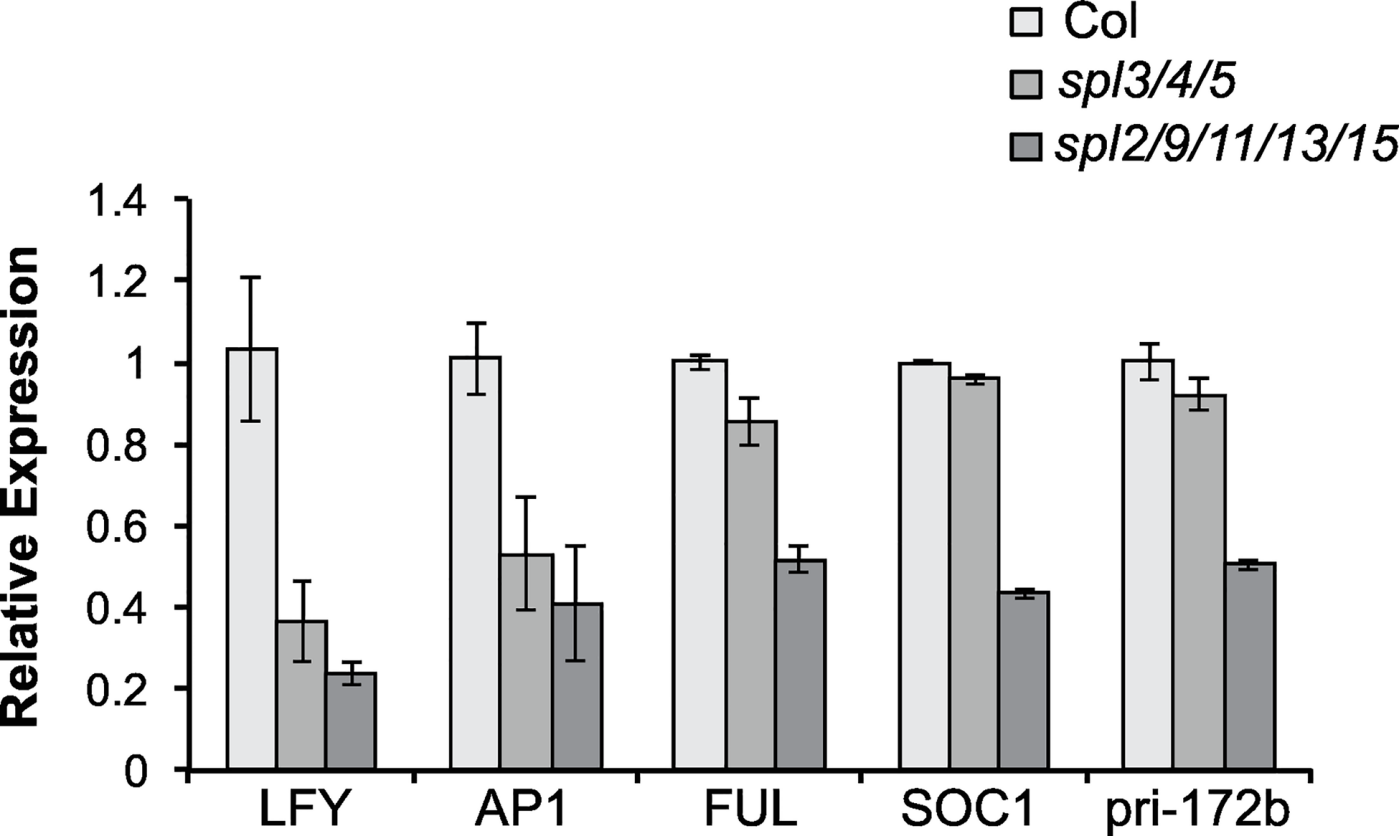
*spl* mutations reduce the expression of genes involved in floral induction and floral meristem identity. qRT-PCR analysis of transcripts isolated from shoot apices of 11 day-old plants. Values are normalized to Col, and represent the mean from 2 biological replicates ± SE.

We explored the molecular basis for these phenotypes by examining the effect of *spl2/9/11/13/15* and *spl3/4/5* on the expression of genes that regulate flowering time (*SOC1*, *MIR172B*) and the floral meristem identity transition (*LFY*, *AP1*, *FUL*) in the shoot apices of 11 day-old shoots grown in LD. *spl2/9/11/13/15* reduced the abundance of *LFY*, *AP1*, *FUL*, and *SOC1* transcripts by about 50%, whereas *spl3/4/5* reduced the expression of *LFY* and *AP1*, but had little effect on the expression of *FUL* or *SOC1* (Fig 6). This result is consistent with the developmental phenotypes of these genotypes, and indicates that *SPL2*, *SPL9*, *SPL11*, *SPL13* and *SPL15* promote both floral induction and floral meristem identity, whereas *SPL3*, *SPL4* and *SPL5* primarily promote floral meristem identity.

**Fig 7:**
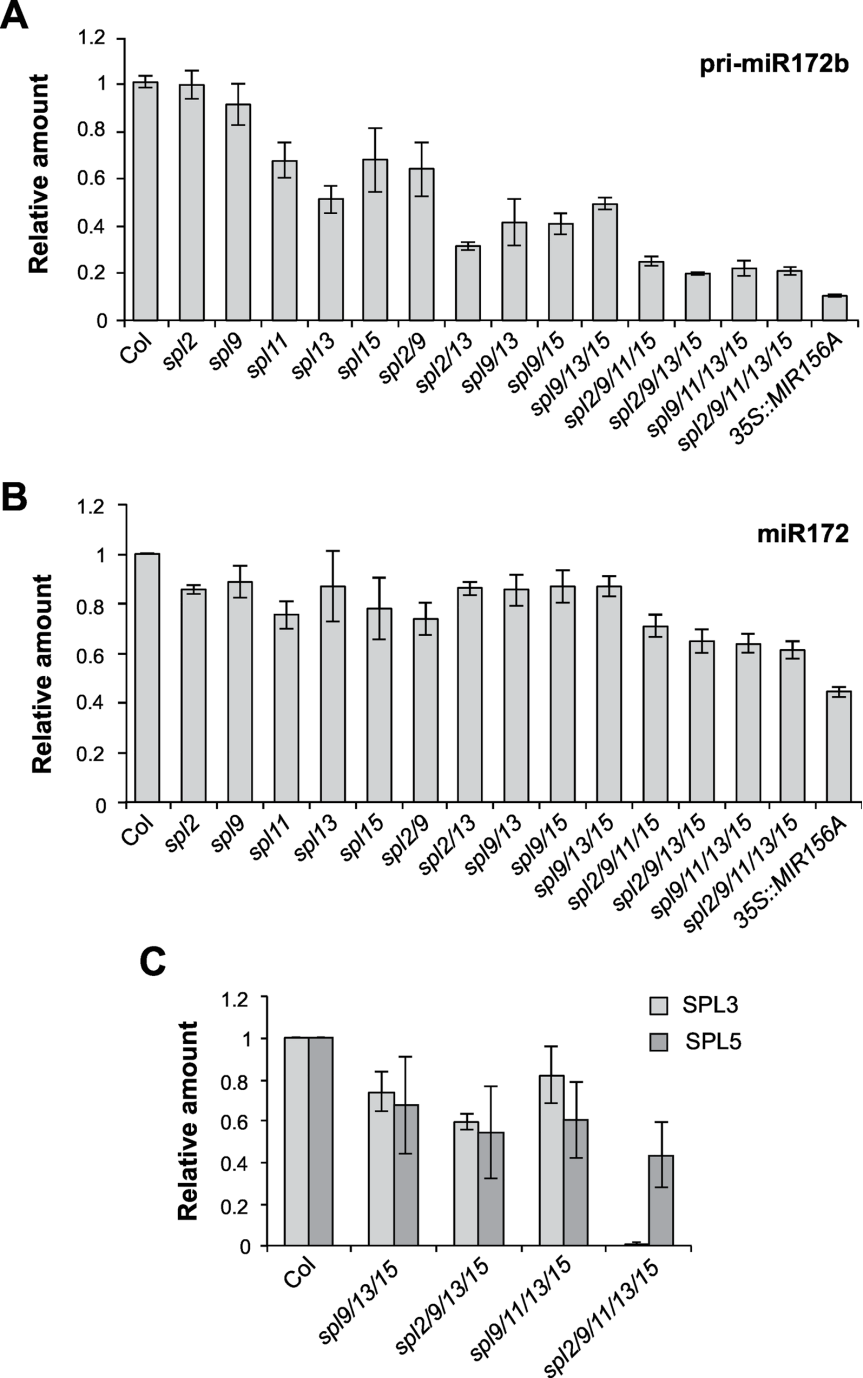
miR172 levels are reduced in *spl* mutants. The abundance of (A) pri-miR172b, (B) miR172 and (C) *SPL3* and *SPL5* mRNA in the shoot apices of 16-day-old *spl* mutants. Values are the average of 3 biological replicates, ± SE.

miR156 is thought to repress floral induction by repressing the expression of miR172 [7], thus elevating the expression of miR172-regulated AP2-like transcription factors, which repress the expression of floral activators, such as *FT* and *SOC1* [48–50]. miR156 modulates the level of miR172 through its effect on the expression *MIR172B* [51]. *MIR172B* is a direct target of SPL9 [7]. To determine if other *SPL* genes also regulate the expression of *MIR172B*, we examined the abundance of pri-miR172b and miR172 in the shoot apices of 16-day old *spl* mutants grown in SD. *spl2* and *spl9* had normal levels of pri-miR172b, but *spl11*, *spl13* and *spl15* all had slightly reduced amounts of this transcript (Fig 7A). *spl2/13*, *spl9/13*, *spl9/15*, and *spl9/13/15* all had between 30–50% of the normal amount of pri-miR172b, and higher order mutants had approximately 20% of the normal pri-miR172b levels (Fig 7A). In contrast to their effect on pri-miR172b, most of these mutant lines only produced a small decrease in the level of miR172 (Fig 7B), possibly because of feedback regulation of other miR172 genes by the AP2-like transcription factors targeted by this miRNA [48]. The biggest decrease in miR172 was observed in *spl2/9/11/1315*, which had approximately 60% of the normal level of this transcript. Thus, *SPL2*, *SPL9*, *SPL11*, *SPL13* and *SPL15* all promote the expression of *MIR172B*, and all of these genes must be repressed to produce a significant reduction in the level of miR172.

To determine if the effect of these mutations on miR172 is functionally significant, we examined the expression of *SPL3* and *SPL5*. These *SPL* genes are downstream of miR172 and are repressed by the miR172-regulated AP2-like transcription factors, TOE1 and TOE2 [7,51]. *SPL3* and *SPL5* transcripts were reduced by approximately 30–40% in *spl9/13/15*, *spl2/9/13/15*, and *spl9/11/13/15*, and by an even greater amount in *spl2/9/11/13/15* (Fig 7C). These mutants had similar effects on miR172 and *SPL5* transcripts, but *SPL3* transcripts were present at a much lower level in *spl2/9/11/13/15* than was expected from the effect of this genotype on miR172. At present, we have no explanation for this effect. In any case, these results suggest that in addition to directly targeting the transcripts of *SPL3*, *SPL4* and *SPL5*, miR156 represses the transcription of these genes by elevating the expression of AP2-like transcription factors.

### miR156-regulated *SPLs* repress adventitious root development

miR156-regulated *SPL* genes have been reported to repress lateral root development in *Arabidopsis* [31]. To explore the function of *SPL* genes in root development, we examined the expression of the rSPL and sSPL reporters in the roots of 12-day-old plants. With the exception of rSPL4 and rSPL5, all rSPL reporters were expressed in the root (S7 Fig). rSPL2 and rSPL11 were most strongly expressed at the root tip, rSPL3 was strongly expressed outside the root tip, rSPL15 was expressed most strongly in the stele of the primary root, and rSPL6, rSPL9, rSPL10 and rSPL13 were expressed in both the root tip and in more mature parts of the root. In contrast, the only miR156-sensitive constructs that were expressed in the root were sSPL6, sSPL9 and sSPL11. sSPL6 was expressed in the same pattern as rSPL6, but at a lower level. sSPL9 was expressed in the stele of the primary root, but not in lateral roots, whereas sSPL11 was expressed exclusively at the tip of the primary and lateral roots. Thus, most *SPL* genes are transcribed in roots but their expression is strongly repressed by miR156 for at least two weeks after germination.

**Fig 8:**
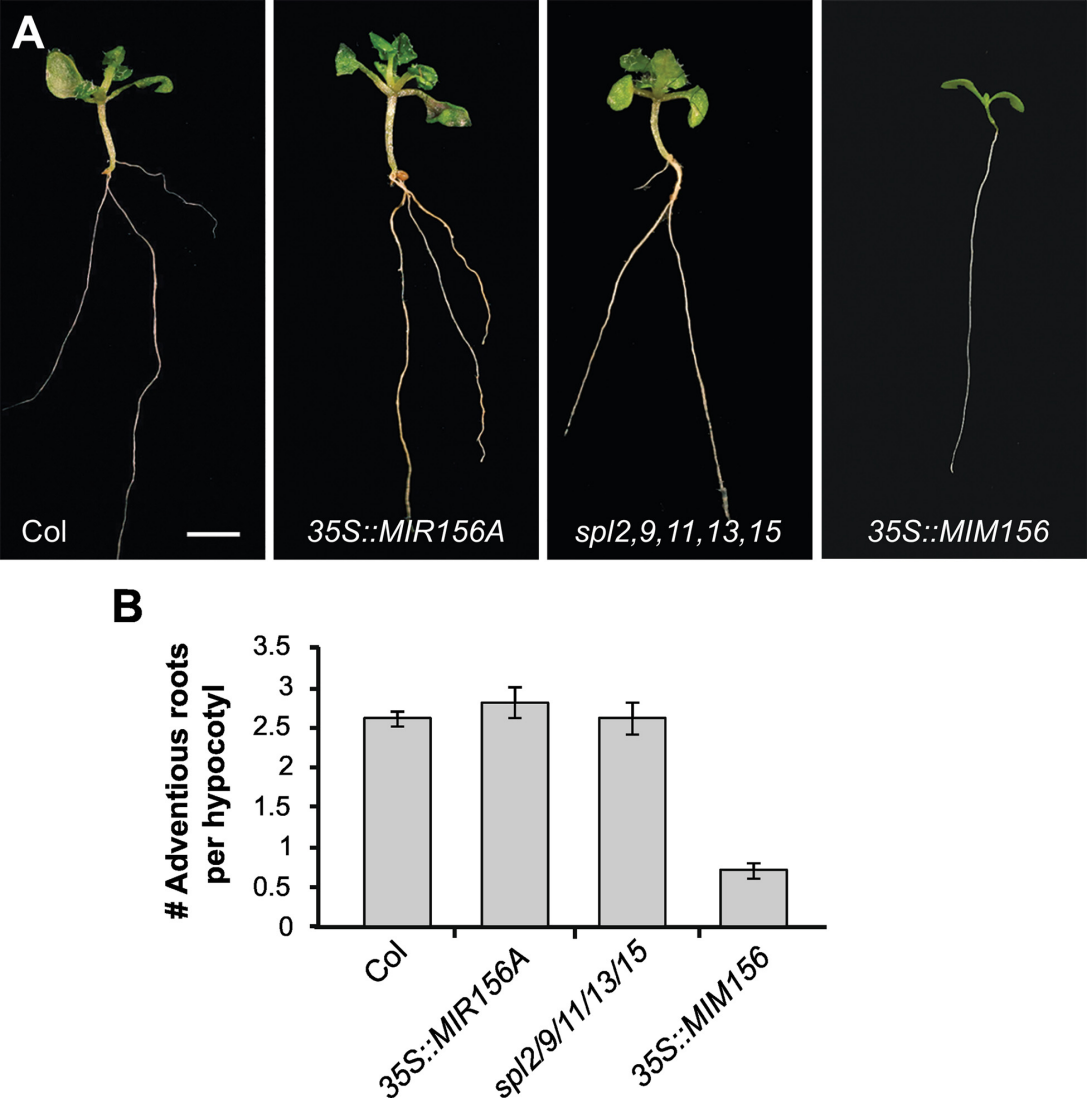
Elevated *SPL* expression inhibits adventitious root production. (A) Roots originating from the hypocotyl of Col, transgenic, and *spl* mutant seedlings after removal of the primary root. (B) The number of adventitious roots produced by the genotypes illustrated in (A). * Significantly different from Col, p<0.05.

Variation in the capacity for adventitious root production is often used as a marker of shoot maturation in woody plants; in general, cuttings from juvenile nodes root more readily than cuttings from adult nodes [52]. To determine if *SPL* genes regulate adventitious root development, we removed the root system of wild-type and mutant seedlings in order to induce root production from the base of the hypocotyl (Fig 8A). Plants transformed with *35S*:*MIR156A*, as well as hypocotyls of the *spl2/9/11/13/15* mutant, produced the same number of adventitious roots as wild-type plants (Fig 8B). This is not surprising because miR156 is already present at very high levels in young seedlings, and *SPL* gene expression is strongly repressed during this phase (Fig 2). In contrast, plants transformed with *35S*::*MIM156*—which causes an increase in *SPL* expression [53]—produced significantly fewer adventitious roots than wild-type plants (Fig 8B). We conclude that miR156-targetted *SPL* genes inhibit adventitious root development.

## Discussion

Our results demonstrate that the effect of miR156 on shoot development can be largely, if not completely, explained by its effect on the expression of the 10 *SPL* genes that have targets sites for this miRNA. Six of these genes—*SPL2*, *SPL9*, *SPL10*, *SPL11*, *SPL13* and *SPL15*—regulate many different aspects of vegetative and reproductive development, and have overlapping, but subtly distinct roles in these processes. Of these, *SPL9*, *SPL13*, and *SPL15* are the most important. Loss-of-function mutations in *SPL9* and *SPL13* are the only single gene mutations that had a significant effect on shoot development, and *spl9*, *spl13*, and *spl15* interacted more strongly with each other than with any other *spl* mutations. Previous genetic analyses have indicated that *SPL9* and *SPL15* play significant roles in shoot development [7,19,23,26,27,33,54], but this is the first evidence that *SPL13* also plays a major role in this process. In contrast to these six genes, we found that *SPL3*, *SPL4* and *SPL5* are not required for vegetative morphogenesis or floral induction. This is at odds with the conclusions of several previous studies [3,32,51,55,56], which employed transgenic lines constitutively expressing miR156-resistant versions of these genes. Our results suggest that the phenotype of these transgenic lines is an artefact of the extremely high level of *SPL3*, *SPL4* or *SPL5* expression, and does not reflect the true function of these genes. The loss-of-function phenotype of the *spl3/4/5* triple mutant indicates that these genes are required after floral induction for the transformation of the vegetative meristem into an inflorescence meristem. Finally, we showed that SPL genes repress the production of adventitious roots, providing an explanation for the long-standing observation that rooting ability declines with the age of the shoot.

### The function of miR156

In our original analysis of vegetative phase change in *Arabidopsis* [57], we found that juvenile leaves can be divided into two classes based on their morphology and their sensitivity to gibberellin. Specifically, we found that the identity of the first two rosette leaves is distinct from, and much more stable than, the identity of subsequent juvenile leaves. The results presented here provide a molecular explanation for this observation. We found that miR156 is expressed at very high levels in leaves 1 and 2 and completely blocks the expression of all *SPL* genes in these leaves. As the level of miR156 declines in subsequent leaves, the expression of some *SPL* genes increases significantly while others remained strongly repressed. During this latter phase, factors that promote the expression of SPL genes, such as GA [54,56] or floral inductive signals [11,58], are capable of increasing *SPL* activity above the threshold set by miR156, enabling phase transitions to occur. During this latter phase, miR156 may act to fine-tune the expression of some of its targets (e.g. *SPL3*, *SPL9*, *SPL13* and *SPL15*), and to set a threshold for the expression of other targets (e.g. *SPL2*, *SPL10* and *SPL11*). This latter function would ensure that only factors that strongly promote *SPL* transcription produce functionally significant changes in *SPL* activity. This might be important for preventing transient increases in *SPL* activity from prematurely promoting floral induction, for example. Our results indicate that miR156 does not play a direct role in floral induction because the abundance of miR156 does not change significantly during this process. However, miR156 could regulate this process indirectly, by ensuring that floral induction only occurs under appropriate environmental conditions.

miR156 represses *SPL* gene expression by cleaving *SPL* transcripts [3,59–61] and by promoting their translational repression [8,62,63], but the relative importance of these activities is still unknown. It is therefore significant that the steep decline in miR156 levels early in shoot development is not accompanied by a corresponding increase in *SPL* transcripts. This could either mean that miR156 represses *SPL* expression primarily by translational repression, or that the amount of miR156 is sufficient to maximally induce the cleavage of most SPL transcripts, even when this miR156 is present at relatively low levels. However, this latter explanation implies that variation in the abundance of miR156 is functionally irrelevant because it does not produce a change in gene expression, and this is not the case; GUS expression from the miR156-sensitive reporters for *SPL3*, *SPL9*, and *SPL13* increased substantially during shoot development in a miR156-dependent fashion. We interpret the relatively small increase in *SPL* transcript levels as evidence that miR156 regulates the expression of most of its targets primarily through its effect on translation, rather than via its effect on transcript stability.

### The function of miR156-regulated SPL genes in vegetative development

*SPL2*, *SPL9*, *SPL10*, *SPL11*, *SPL13*, and *SPL15* have overlapping functions, and together promote vegetative traits associated with the adult phase. Inappropriate expression of any one of these genes early in shoot development results in the precocious expression of traits that are normally expressed later in development, while the combined loss of these genes prolongs the expression of juvenile traits, and produces a phenotype that is essentially indistinguishable from that of plants constitutively expressing miR156. However, the phenotypes of plants lacking subsets of these genes demonstrate that *SPL9*, *SPL13* and *SPL15* play a much larger role in vegetative development than *SPL2*, *SPL10* and *SPL11*. This is at least partly explained by the level at which these genes are expressed in the shoot apex. Both the gain-of-function and the loss-of-function phenotypes of these six genes indicate that they have closely related functions, but *SPL9*, *SPL13* and *SPL15* are much more highly expressed in the vegetative shoot than *SPL2*, *SPL10* and *SPL11*. While the miR156-sensitive constructs for these genes are expressed at very different levels during vegetative development, the miR156-resistant constructs for *SPL2*, *SPL9*, *SPL10*, *SPL11* and *SPL13* are expressed at roughly the same level in the shoot apex. This observation suggests that the differential expression of these genes is due to their differential sensitivity to miR156. In contrast to these genes, *SPL3*, *SPL4* and *SPL5* do not have dramatic effects on vegetative morphology. This is particularly surprising in the case of *SPL3* because it is highly expressed in the rosette. Over-expression and under-expression of miR156 affects the response of *Arabidopsis* to heat stress [21] and salt stress [22,64], and it may be that *SPL3* regulates these physiological processes rather than shoot morphogenesis.

Previously, we suggested [57] that the timing of vegetative phase change is regulated independently of leaf initiation because mutations in *ALTERED MERISTEM PROGRAMMING 1* (*AMP1*) and *PAUSED (PSD*) increase (*amp1*) or decrease (*psd1*) the number of juvenile leaves without changing the timing of vegetative phase change. Instead, the effect of these mutations on juvenile leaf number appeared to be attributable to an increase (*amp1*) or a decrease (*psd*) in the rate of leaf initiation. However, the tight linkage between the timing of vegetative phase change and rate of leaf initiation in plants with elevated or reduced levels of *SPL* gene expression (this report; [17]) suggests that this hypothesis needs to be re-evaluated. In particular, the evidence that *AMP1* promotes miRNA-mediated translational repression [65] raises the possibility that the effect of *amp1* on juvenile leaf number could be attributable to the effect of this mutation on miR156 activity, rather than being an indirect effect of the accelerated rate of leaf initiation in this mutant. On the other hand, the effect of *amp1* on vegetative phase change is inconsistent with its proposed role in miRNA-mediated translational repression, at least with respect to miR156. Mutations that interfere with the activity of miR156, such as *ago1*, *sqn* and *suo*, reduce the number of juvenile leaves [62,66,67], whereas *amp1* has the opposite phenotype [57]. Indeed, the phenotype of *amp1* is more consistent with an increase in miR156 activity than with a decrease in miR156 activity. Further studies will be necessary to determine if the effect of *psd* and *amp1* on vegetative phase change is an indirect result of their effect on leaf initiation, or reflects a more direct role in this process.

### The function of miR156-regulated SPL genes in flowering

In Col, miR156-regulated genes are less important for floral induction than they are for vegetative phase change. Under LD, *35S*::*MIR156A* and *spl2/9/10/11/13/15* plants only flowered 8 days later than normal but produced 22 additional juvenile leaves; under SD, they also flowered 8 days later than normal but produced more than 60 additional juvenile leaves. These genes may be more important for flowering in other ecotypes, however. Col has relatively low levels of the floral repressor, FLC [68], because it possesses a non-functional allele *FRI*, which is required for the expression of *FLC* [69]. In *Arabis alpina* and *Cardamine flexuosa* [70,71], FLC acts together with miR156 to repress flowering; plants in which both of these factors are expressed at high level are extremely late flowering. *Arabidopsis* ecotypes with functional alleles of FRI have relatively high levels of FLC [72,73] and it will be important to determine if miR156-regulated *SPL* genes are more important for floral induction in these ecotypes. The extent to which *SPL* genes are required for floral induction also appears to be strongly dependent on environmental conditions. Both we and Wang et al [18] found that *35S*::*MIR156* had a relatively small effect on flowering time SD, whereas Schwab et al [6] reported that *35S*::*MIR156* flowers at about 7 months in SD. This difference is unlikely to be attributable to variation in the strength of the *35S*::*MIR156* transgenes used in these experiments because the phenotype of our *35S*::*MIR156* line was nearly identical to the *spl2/9/10/11/13/15* mutant, implying that this *35S*::*MIR156* transgene completely, or nearly completely, eliminates SPL activity. This variability suggests that the effect of *35S*::*MIR156* on flowering time under SD is strongly dependent on environmental factors other than photoperiod, such as light quantity and quality, temperature, water availability etc. *Arabidopsis* is extraordinarily sensitive to minor variation in environmental conditions [74], and it may be that *SPL* genes only play a major role in floral induction in Col when all of the environmental factors that positively regulate this process are absent.

**Fig 9:**
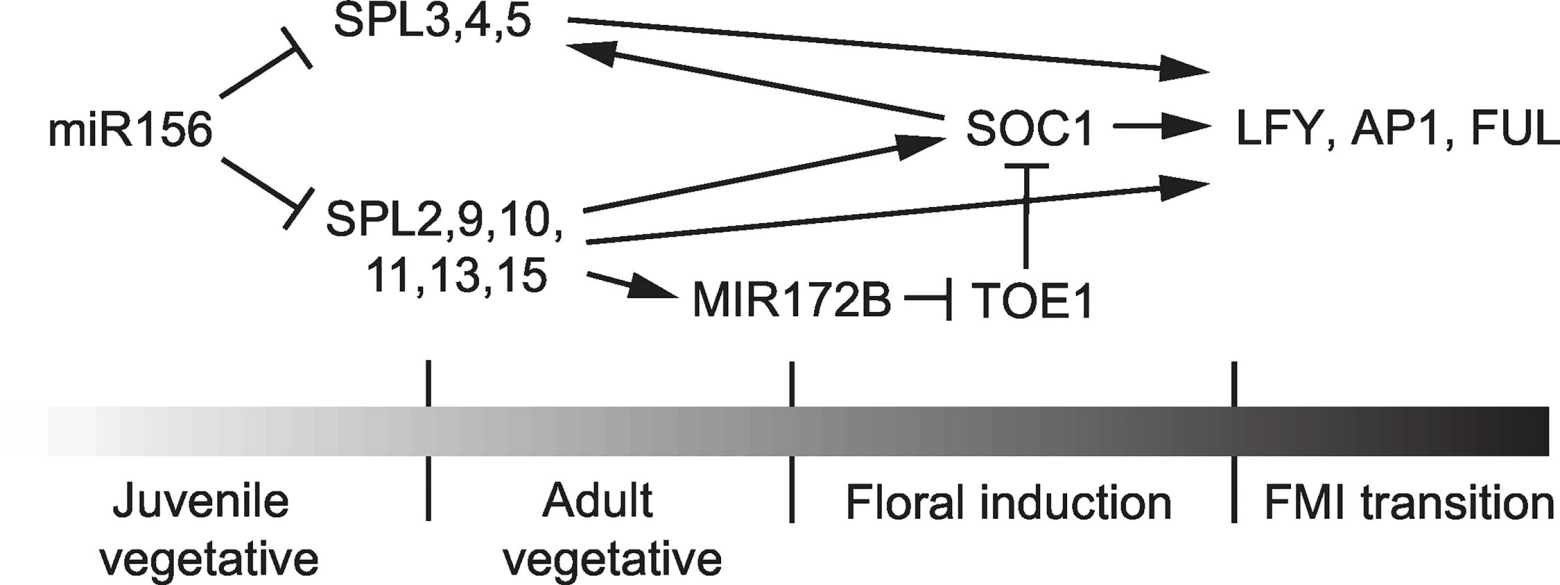
Regulatory interactions between SPL genes and genes involved in floral induction and the floral meristem identity transition. The gray scale symbolizes the level of SPL activity.

A summary of the role of miR156-regulated SPL genes in flowering is shown in Fig 9. Many studies have focused on the role of *SPL3*, *SPL4* and *SPL5* in floral induction because these genes are strongly up-regulated during floral induction and cause early flowering when expressed under the regulation of the constitutive *CaMV 35S* promoter [3,11,32,51,55,56,58]. Although *spl3/4/5* mutants consistently had extra cauline leaves, they displayed little or no delay in flowering time under both LD and SD, and had no effect on the expression of the flowering time genes, *MIR172B* and *SOC1*. This latter observation is consistent with previous studies indicating that *SPL3*, *SPL4* and *SPL5* are downstream of *SOC1*, *miR172*, and the flowering time regulator, *FT* [51,75,76]. The inflorescence phenotype of *spl3/4/5* is explained by the effect of this genotype on the floral meristem identity genes *LFY*, *AP1* and *FUL*. We found that the *spl3/4/5* triple mutant has reduced levels of the transcripts of these three genes. This is consistent with previous studies showing that *LFY*, *AP1* and *FUL* transcripts are elevated in plants over-expressing SPL3, and with the evidence that SPL3 binds to the promoters of these three genes [16,18,51,56]. As is the case with *SPL3*, *SPL4* and *SPL5* [3,32], over-expression of *LFY*, *AP1* and *FUL* accelerates flowering, but loss-of-function mutations in these genes are not late flowering [41,77–80]. These and many other studies demonstrate that floral induction is distinct from the floral meristem identity transition. Floral induction involves changes in many different aspects of shoot development including the growth and morphogenesis of rosette leaves, stem elongation, and a change the identity of the lateral organs produced by the shoot apical meristem [81,82]. The floral meristem identity transition is the latter of these processes [83]. Over-expression of genes involved in the floral meristem identity transition, such as *LFY* or *AP1*, forces the vegetative meristem to become an inflorescence meristem, resulting in early flowering. Similarly, over-expression of *SPL3*, *SPL4*, and *SPL5* accelerates the floral meristem identity transition, but it is apparent from their loss-of-function phenotype that these genes do not play a general role in floral induction. Our results are consistent with the observation that the ortholog of *SPL3/4/5* in *Antirrhinum majus*, *SBP1*, acts after floral induction to promote the floral meristem identity transition [84].

Other miR156-regulated SPL genes are required for both floral induction and floral meristem identity (Fig 9). As in vegetative phase change, *SPL9*, *SPL13* and *SPL15* play dominant roles in both of these processes, but *SPL2*, *SPL10* and *SPL11* also make important contributions, particularly in SD. This is evident from the observation that flowering was only delayed significantly in SD in genotypes that lacked *SPL9*, *SPL15*, and either *SPL2* or *SPL11*. *SPL2* and *SPL11* also contribute to floral induction in LD, but their effect is relatively modest under these conditions. SPL9 is bound to the promoters of the flowering time genes *MIR172B* and *SOC1 in vivo*, and promotes their expression when it is over-expressed [7,18]. Although loss of *SPL9* does not have a major effect on the expression of *MIR172B* and *SOC1*, their expression was significantly reduced in *spl2*,*9*,*11*,*13*,*15* mutants. Together, these results suggest that SPL2, SPL9, SPL11, SPL13 and SPL15 directly promote the transcription of these genes. The possibility that SPL2, SPL9, SPL11, SPL13 and SPL15 promote *MIR171B* and *SOC1* transcription by a different mechanism should also be considered. For example, SPL9 blocks the dimerization of the TCP4 and CUC2 transcription factors by binding to TCP4 [85], and there is evidence that it regulates the response of plants to GA by interfering with the activity of the DELLA protein, RGA [54]. However, if SPL2, SPL9, SPL11, SPL13 and SPL15 act primarily by modulating the activity of other transcription factors, one would expect the dimerization domain in these functionally redundant proteins to be highly conserved, and this is not the case. SPL9 interacts with TCP4 [85] and RGA [54] via its C-terminal region, and this region is highly variable between SPL2, SPL9, SPL11, SPL13 and SPL15. The most highly conserved region of these proteins is their DNA-binding domain, the SBP-box. For this reason, we suspect that these SPL proteins act primarily as direct transcriptional activators or repressors.

### The function of SPL genes in root development

Adventitious root production is increased in plants with elevated levels of miR156, such as the *Teopod/Corngrass* mutants of maize [86] or tobacco transformed with *35S*::*miR156* [87], suggesting that SPL genes normally inhibit this process. We found that elevated levels of miR156 have no effect on adventitious root production in the hypocotyl, but reducing miR156 activity inhibits this process, implying that SPL proteins inhibit adventitious root production, just as they inhibit lateral root production in the primary root [31]. *SPL* expression increases in successive nodes of woody plants [9], so this result may provide an explanation for the correlation between shoot age and the loss of rooting capacity in these plants [52,88]. Unfortunately, we were unable to investigate whether variation in the capacity for adventitious root production is a marker for vegetative phase change or reproductive phase change because the short internodes of an *Arabidopsis* rosette make it difficult to examine adventitious root production at different stages of vegetative development. *SPL* gene expression increases during both vegetative phase change and floral induction, and there may be a threshold level of *SPL* gene expression required to repress adventitious root development. This is an important question to answer because rooting ability determines the ease with which many horticulturally-important species can be propagated. The ability to *SPL* expression using exogenous factors could be of considerable practical importance.

*SPL* genes arose early in plant evolution, and are present in multiple copies in all land plants examined to date [15,89]. We focused on the roles of miR156-regulated *SPL* genes in shoot and root morphogenesis, but these genes are involved in many other aspects of plant biology as well. The reporter lines and mutant stocks described here will be useful for defining the full range of their function, and the role of miR156 in sculpting their activity.

## Materials and Methods

### Plant material and growth conditions

All of the stocks used in this study were in a Col genetic background. Mutations that were originally generated in a different genetic background (*spl3-1*, *spl11-1*, *spl13-1*, *spl13-2*, *spl13-3*) were crossed to Col 6 or more times. *spl2-1* (SALK_022235), *spl3-1* (FLAG_173C12), *spl4-1* (CS90956), *spl4-2* (CS88228), *spl4-3* (CS96315), *spl5-1* (SAIL_265_D02), *spl6-1* (CS90560), *spl6-2* (CS93521), *spl6-3* (CS92990), *spl9-4* (SAIL_150_B05), *spl11-1* (FLAG_422H07) and *spl15-1* (SALK_074426) were obtained from the *Arabidopsis* Biological Resource Center (Ohio State University, Columbus, OH). *spl13-1* (line 2754), *spl13-2* (line 3697) and *spl13-3* (line 6746) were obtained from the TILL*er* service (Carlos Alonso Blanco, Centro Nacional de Biotecnologia, Madrid, Spain). Seeds were sown on Farfard #2 potting soil, placed at 4°C for 2 to 3 days, and grown at 22°C in Conviron growth chambers under either long days (16 hrs light/8 hrs. dark; 95 μmol m^-2^ s^-1^) or short days (10 hrs. light/ 14 hrs. dark; 180 μmol m^-2^ s^-1^) using a 5:3 combination of white (USHIO F32T8/741) and red-enriched (Interlectric F32/T8/WS Gro-Lite) fluorescent lights. As indicated in the Results, one experiment was performed with plants growing in at 8 hrs. light/ 14 hrs. dark; 180 μmol m^-2^ s^-1^). Plant age was measured from the date seeds were transferred to the growth chamber.

For analyses of root development, plants were grown on agar in petri dishes on 1/2 strength Murashige and Skoog medium under long day conditions. Sugar was omitted from the medium because it affects the expression of miR156 [90,91].

### Transgenic plants

The miR156-sensitive and miR156-resistant SPL-GUS fusion lines were constructed by placing the *GUS* gene from pCAMBIA3301 or the *GUS+* gene from pCAMBIA1305, at the 5' or 3' end of the coding sequence of different SPL genes (S1 Fig). In all but two cases, the construct consisted of the genomic sequence extending from gene upstream of the *SPL* gene to the gene downstream of the *SPL* gene. The only exceptions were *SPL10* and *SPL11*, which were constructed according to the strategy described in Yang et al [92], and only extend to the end of the coding region. These constructs were inserted into pCAMBIA3300 or pCAMBIA3301 [93] and then transformed into Col by floral dipping [94]. The primers used in making these constructs are listed in S4 Table. Transgenic plants were identified using Basta resistance, and their T2 progeny were screened to identify lines segregating 3:1 for the transgene. The T3 progeny of T2 plants were then screened to identify lines homozygous for the insertion.

### GUS staining and histology

Plants were fixed in 90% acetone on ice for 10 minutes, and were washed with GUS staining buffer (4mM potassium ferrocyanide and 4mM potassium ferricyanide in 0.1 M PO_4_ buffer), and stained overnight at 37° in 2mM X-Gluc in GUS staining buffer. *in situ* hybridization was performed on 21-day old plants, which were processed according to Xu *et al* [46].

### CRISPR-cas9 induced mutations

The *spl10* CRISPR-cas9 mutant lines were generated with the guide RNA (5’-GGT ACC TCG AGA GCT GTG GA-3’) using protocols described previously [95,96]. Primers flanking the guide RNA (5’-AGG ACA AAC GAT GCA ATC TTG-3’, 5’-TTT TCT TCC GAG CAA CAA CAG-3’) were used to verify the mutations in the *spl10-2*, *spl10-3* and *spl10-4* alleles.

### qPCR analysis of transcript levels

RNA was extracted from the shoot apices of plants grown under SD or LD conditions, as indicated in the text. Shoot apices were harvested by removing the cotyledons and all leaves larger that 5 mm. Total RNA was isolated using Trizol (Invitrogen), and was then treated by RNase-free DNase (Ambion) following the manufacturer's instructions. 600 ng RNA was used for reverse transcription of miR156, miR272 and SnoR101, using miR156, miR172 and SnoR101-specific RT primers. Quantification of miR156 and miR172 was performed according to [97]. Quantification of miR172b was performed according to [7]. qPCR reactions were run in triplicate and the results were averaged to produce the value for 1 biological replicate; the data presented here are the average of 2–4 biological replicates. Primers used for RT-PCR and qRT-PCR are listed in S4 Table.

### Adventitious root induction

Plants were grown on 1/2 MS under LD conditions for 6 days, and primary roots were then removed with a scalpel. Plants were then etiolated for 2 days in darkness and returned to LD conditions. Adventitious roots were analysed 7 days after return to LD conditions.

## Supporting Information

S1 Fig The structure of *rSPL* and *sSPL* reporter constructs. (PDF)

S2 Fig Expression of rSPL and sSPL reporters in the inflorescence. (PDF)

S3 Fig The effect of *rSPL3* transgenes on the abundance of the *SPL3* transcript. (PDF)

S4 Fig RT-PCR analysis of the effect of *spl* mutations on the abundance of the mutant transcript. (PDF)

S5 Fig q-PCR analysis of *SPL9* and *SPL13* copy number in different ecotypes. (PDF)

S6 Fig The rate of leaf initiation in *spl3/4/5* mutants. (PDF)

S7 Fig Expression of rSPL and sSPL reporters in the root. Scale bar = 2mm.
(PDF)

S1 Table Sequence of the miR156 target site in miR156-sensitive and miR156-resistant *SPL-GUS*. (PDF)

S2 Table Flowering time of plants over-expressing *spl4* mutations. (PDF)

S3 Table The phenotype of loss-of-function alleles of miR156-regulated *SPL* genes in LD. (PDF)

S4 Table PCR primers used in this study. (XLSX)
