# Supplementary material for: Developmental Functions of miR156-Regulated SQUAMOSA PROMOTER BINDING PROTEIN-LIKE (SPL) Genes in Arabidopsis thaliana
Source: PLoS Genet. 2016 Aug 19;12(8):e1006263. doi: 10.1371/journal.pgen.1006263 (PMC4991793; doi:10.1371/journal.pgen.1006263)
Supplement: S1 Fig — (PDF) [file pgen.1006263.s001.pdf]

S1 Fig

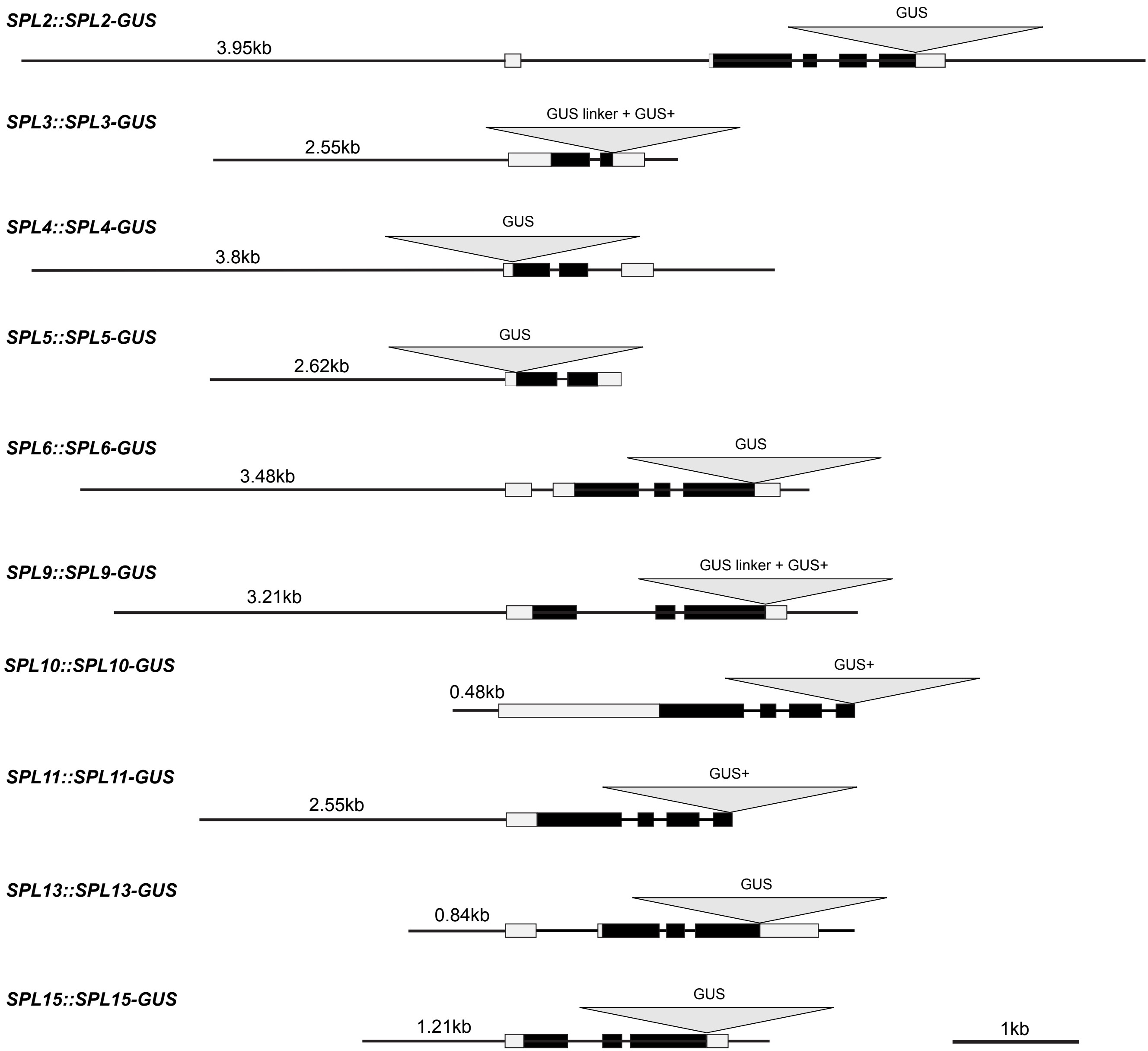
SPL2::SPL2-GUS
GUS
3.95kb
SPL3::SPL3-GUS
GUS linker + GUS+
2.55kb
SPL4::SPL4-GUS
GUS
3.8kb
SPL5::SPL5-GUS
GUS
2.62kb
SPL6::SPL6-GUS
GUS
3.48kb
SPL9::SPL9-GUS
GUS linker + GUS+
3.21kb
SPL10::SPL10-GUS
GUS+
0.48kb
SPL11::SPL11-GUS
GUS+
2.55kb
SPL13::SPL13-GUS
GUS
0.84kb
SPL15::SPL15-GUS
GUS
1.21kb
1kb
