# Supplementary figures and images for: Developmental Functions of miR156-Regulated SQUAMOSA PROMOTER BINDING PROTEIN-LIKE (SPL) Genes in Arabidopsis thaliana
Source: PLoS Genet. 2016 Aug 19;12(8):e1006263. doi: 10.1371/journal.pgen.1006263 (PMC4991793; doi:10.1371/journal.pgen.1006263)

S2 Fig

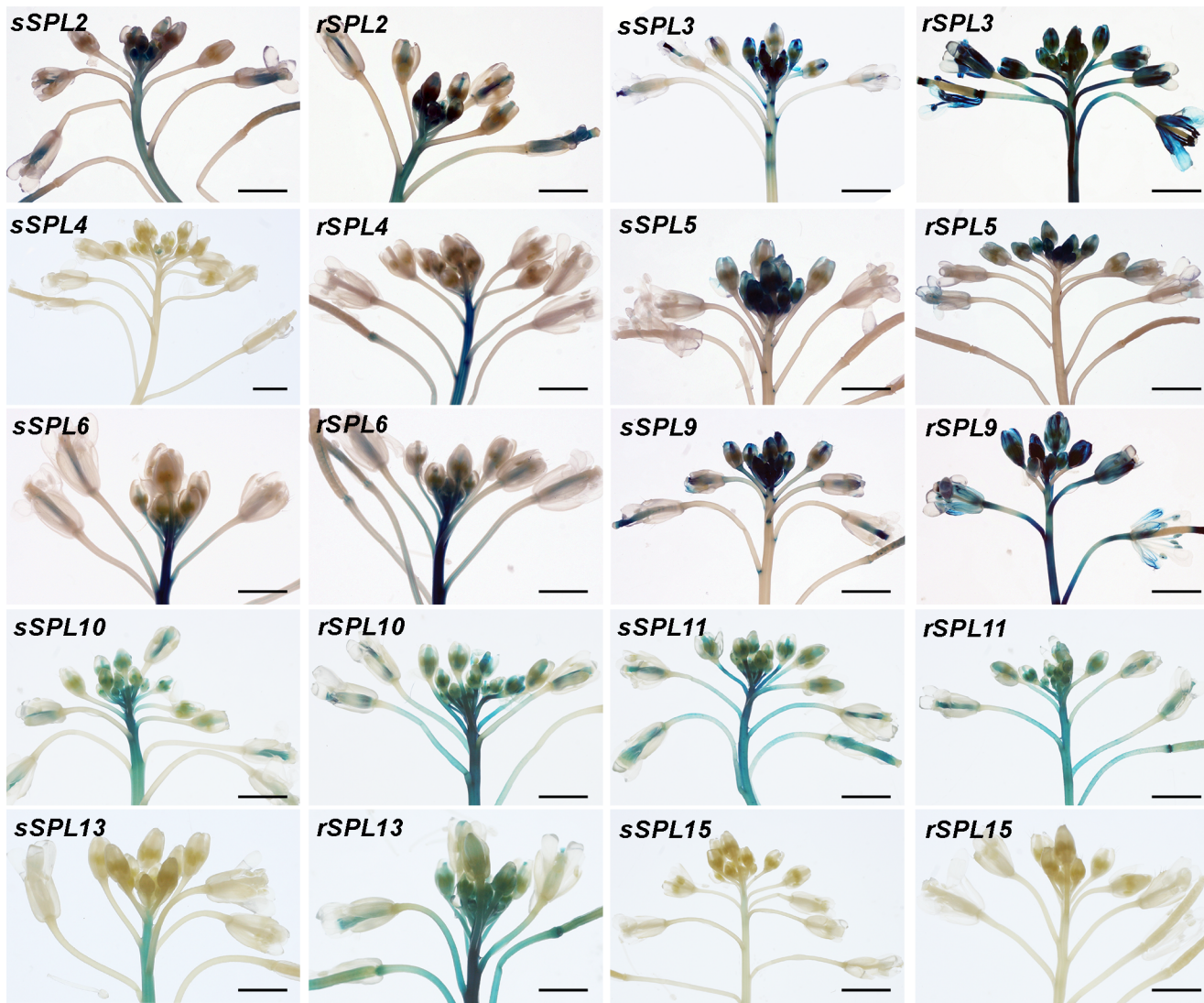

Supplement: S2 Fig — (PDF) [file pgen.1006263.s002.pdf]

S3 Fig

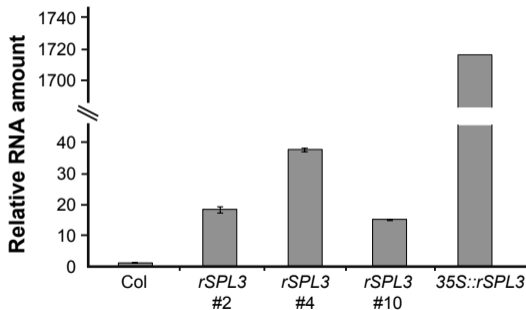

Supplement: S3 Fig — (PDF) [file pgen.1006263.s003.pdf]

# S4 Fig

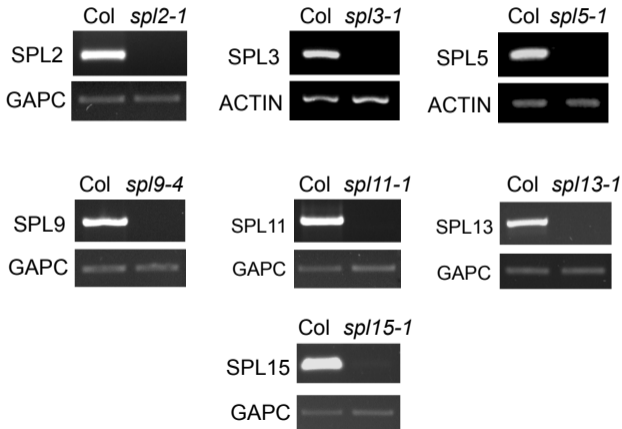

Supplement: S4 Fig — (PDF) [file pgen.1006263.s004.pdf]

S5 Fig

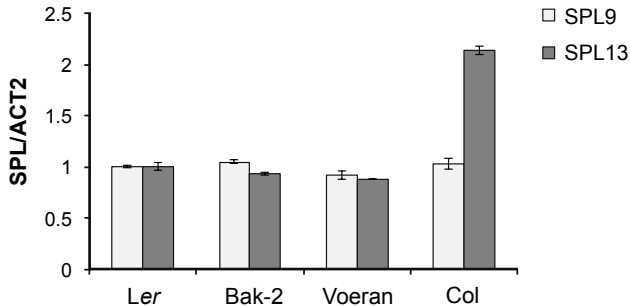

Supplement: S5 Fig — (PDF) [file pgen.1006263.s005.pdf]

S6 Fig

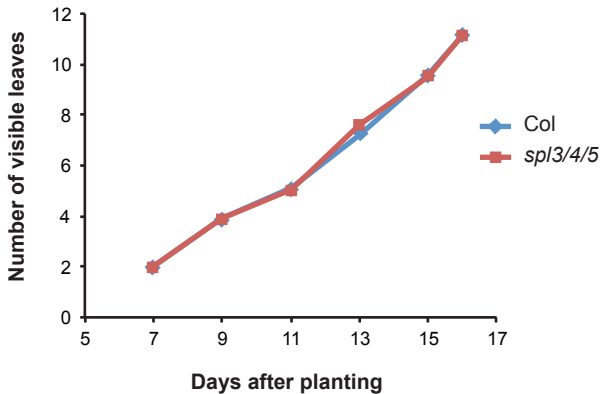

Supplement: S6 Fig — (PDF) [file pgen.1006263.s006.pdf]

# S7 Fig

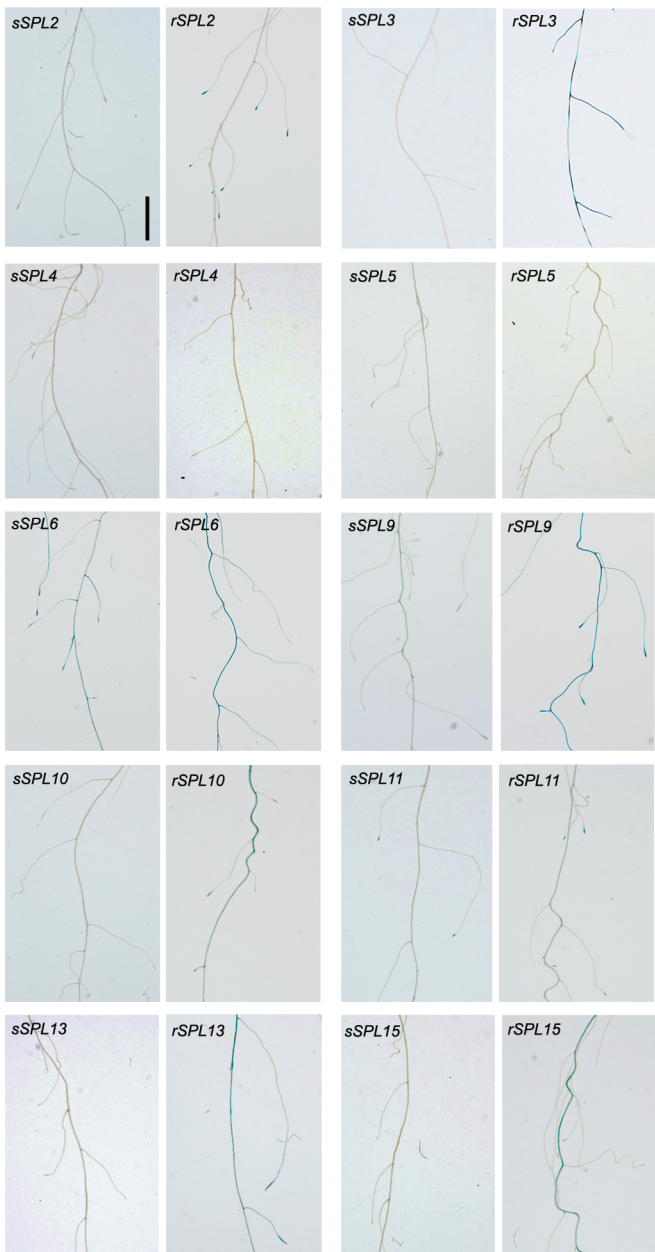

Supplement: S7 Fig — Scale bar = 2mm. (PDF) [file pgen.1006263.s007.pdf]
