# Supplementary material for: Developmental Functions of miR156-Regulated SQUAMOSA PROMOTER BINDING PROTEIN-LIKE (SPL) Genes in Arabidopsis thaliana
Source: PLoS Genet. 2016 Aug 19;12(8):e1006263. doi: 10.1371/journal.pgen.1006263 (PMC4991793; doi:10.1371/journal.pgen.1006263)
Supplement: S1 Table — (PDF) [file pgen.1006263.s008.pdf]

S1 Table. miR156-target sites in SPL-GUS reporters

	Wild-type (miR156-sensitive)	miR156-resistant
SPL2	gtgctctctcttctgtca	gagcactttcactcctttct
SPL3	ttgcttactctcttctgtca	tcagatgtgacgactgcatc
SPL4	ctgctctctctcttctgtca	ccagatgtgacgactgcatc
SPL5	ccgctctctctcttctgtca	ccagatgtgacgactgcatc
SPL6	gtgctctctctcttctgtca	gcgccctatccttgctttcc
SPL9	gtgctctctctcttctgtca	gcgcattgagcttgttaagc
SPL10	gtgctctctctcttctgtca	gcgcactgtcactactctct
SPL11	gtgctctctctcttctgtca	gagcactgtcactactctct
SPL13	gtgctctctctcttctgtca	gcgcattgtcgctccttagc
SPL15	gtgctctctctcttctgtca	gtgcactttcactcctttct
