# Supplementary material for: Developmental Functions of miR156-Regulated SQUAMOSA PROMOTER BINDING PROTEIN-LIKE (SPL) Genes in Arabidopsis thaliana
Source: PLoS Genet. 2016 Aug 19;12(8):e1006263. doi: 10.1371/journal.pgen.1006263 (PMC4991793; doi:10.1371/journal.pgen.1006263)
Supplement: S2 Table — (PDF) [file pgen.1006263.s009.pdf]

S2 Table: The phenotype of loss-of-function alleles of miR156-regulated *SPL* genes in LD

	Juvenile leaves	Rosette leaves	Cauline leaves	Days to flower	n
**Experiment 1**					
Col	4.6 ± 0.1	10.8 ± 0.1	N.D.	26.3 ± 1.0	18
spl9-2	6.2 ± 0.2[a]	12.3 ± 0.1[a]	N.D.	24.4 ± 0.6	22
spl9-4	6.2 ± 0.1[a]	13.2 ± 0.2[a]	N.D.	24.7 ± 0.8	22
spl15-1	5.3 ± 0.1	10.7 ± 0.1	N.D.	27.5 ± 0.9	22
spl15-2	5.0 ± 0.1	11.8 ± 0.1[a]	N.D.	25.7 ± 1.0	21
**Experiment 2**					
Col	5.1 ± 0.1	11.3 ± 0.3	3.0 ± 0.1	26.9 ± 0.3	*23*
spl10-2	4.9 ± 0.1	11.0 ± 0.2	3.0 ± 0.1	25.7 ± 0.2	*23*
spl10-4	5.0 ± 0.1	11.0 ± 0.2	3.1 ± 0.1	25.9 ± 0.2	*23*
spl11-1	6.2 ± 0.2[a]	11.8 ± 0.3	3.5 ± 0.2[a]	26.8 ± 0.4	*22*
spl10-3/spl11-1	6.4 ± 0.2[a]	12.8 ± 0.2[a]	3.8 ± 0.1[a]	26.9 ± 0.2	*24*
**Experiment 3**					
Col	4.6 ± 0.1	10.3 ± 0.2	2.8 ± 0.1	26.0 ± 0.3	*22*
spl13-1	5.5 ± 0.2[a]	11.5 ± 0.3[a]	2.7 ± 0.1	26.6 ± 0.2	*23*
spl13-2	5.6 ± 0.2[a]	10.8 ± 0.3	2.6 ± 0.1	26.5 ± 0.2	*23*
spl13-3	5.5 ± 0.1[a]	10.9 ± 0.2	2.9 ± 0.1	25.2 ± 0.3	*23*
spl9-4/spl15-1/spl13-1	14.8 ± 0.2[ab]	22.3 ± 0.5[a]	3.1 ± 0.1	30.8 ± 0.3[a]	*40*
spl9-4/spl15-1/spl13-3	14.8 ± 0.1[ab]	20.6 ± 0.3[a]	2.5 ± 0.1	29.3 ± 0.2[a`]	*22*
**Experiment 4**					
Col	4.6 ± 0.1	11.0 ± 0.3	3.0 ± 0.1	28.0 ± 0.3	*23*
spl2-1	5.0 ± 0.1	11.2 ± 0.3	2.9 ± 0.1	27.7 ± 0.4	*21*
spl9-4	7.3 ± 0.1[a]	13.0 ± 0.2[a]	3.0 ± 0.1	28.0 ± 0.3	*23*
spl11-1	5.4 ± 0.2[a]	11.2 ± 0.3	3.5 ± 0.2	26.8 ± 0.4	*21*
spl13-1	6.4 ± 0.2[a]	12.2 ± 0.3[a]	2.8 ± 0.1	28.0 ± 0.4	*22*
spl15-1	4.4 ± 0.1	11.1 ± 0.3	2.7 ± 0.1	26.3 ± 0.4	*21*
spl2 spl9	7.8 ± 0.2[a]	13.2 ± 0.5[a]	2.7 ± 0.2	28.2 ± 0.5	*21*
spl2 spl13	6.6 ± 0.2[a]	11.9 ± 0.3	2.6 ± 0.1	28.3 ± 0.4	*22*
spl2 spl9 spl11	6.8 ± 0.1[a]	11.9 ± 0.2	2.2 ± 0.1	25.8 ± 0.3	*24*
spl13 spl15	5.5 ± 0.1[a]	12.7 ± 0.4[a]	3.5 ± 0.2	28.5 ± 0.5	*24*
spl2 spl13 spl15	7.7 ± 0.2[a]	16.4 ± 0.4[b]	3.1 ± 0.1	29.7 ± 0.3	*23*
spl9 spl15	8.2 ± 0.2[ab]	16.5 ± 0.4[b]	3.2 ± 0.1	30.2 ± 0.4[ab]	*21*
spl2 spl9 spl15	9.2 ± 0.2[ab]	18.8 ± 0.5[c]	3.4 ± 0.1	29.8 ± 0.3[ab]	*24*
spl2 spl9 spl11 spl15	8.4 ± 0.2[ab]	16.3 ± 0.4[b]	2.6 ± 0.1	28.5 ± 0.4	*24*
spl9 spl13	12.9 ± 0.3[bc]	16.6 ± 0.4[b]	2.9 ± 0.1	29.5 ± 0.6[ab]	*21*
spl2 spl9 spl13	13.2 ± 0.3[bc]	16.7 ± 0.3[b]	2.7 ± 0.1	29.0 ± 0.4[ab]	*24*
spl9 spl13 spl15	18.9 ± 0.3[cd]	27.1 ± 0.6[cd]	4.1 ± 0.1[cd]	33.0 ± 0.3[cd]	*23*

[a] significantly greater than Col
[b] significantly greater than parents
[c] significantly greater than *spl9/15*
[d] significantly greater than *spl9/13*
N.D. Not determined
