# Supplementary material for: Developmental Functions of miR156-Regulated SQUAMOSA PROMOTER BINDING PROTEIN-LIKE (SPL) Genes in Arabidopsis thaliana
Source: PLoS Genet. 2016 Aug 19;12(8):e1006263. doi: 10.1371/journal.pgen.1006263 (PMC4991793; doi:10.1371/journal.pgen.1006263)
Supplement: S3 Table — (PDF) [file pgen.1006263.s010.pdf]

S3 Table: **Flowering time of transgenic plants over-expressing miR156-resistant *SPL4*, or point mutations in this gene.**

Lines	Early flowering phenotype: Strong	Medium	Weak	# T1 plants
*35S::SPL4*Δ[a]	39%	50%	11%	28
*35S::SPL4*Δm1[b]	0%	45%	55%	47
*35S::SPL4*Δm2[c]	16%	54%	30%	63
*35S::SPL4*Δm3[d]	4%	60%	37%	104

[a]*SPL4* with no miR156 target site, expressed under the regulation of the CaMV 35S promoter.

[b]*SPL4*Δ with the same point mutation as *spl4-1*

[c]*SPL4*Δ with the same point mutation as *spl4-2*

[d]*SPL4*Δ with the same point mutation as *spl4-3*
